# Bio-inspired material-structure-function integrated additive manufacturing of Al-based metamaterials with surpassing energy absorption

**DOI:** 10.1126/sciadv.aea0430

**Published:** 2025-11-14

**Authors:** Xi He, Gan Li, Lei Zhang, Yuhe Huang, Bingyu Xie, Zhifang Shi, Guanghui Feng, Wenbin Liu, Fucong Lyu, Shuo Wang, Zhengrong Yu, Junhua Luan, Chunlu Zhao, Hongxing Lu, Xiaogang Hu, Qiang Zhu, Jian Lu

**Affiliations:** ^1^ Shenzhen Key Laboratory for Additive Manufacturing of High-performance Materials, Department of Mechanical and Energy Engineering, Southern University of Science and Technology, Shenzhen, China.; ^2^ City University of Hong Kong Matter Science Research Institute (Futian), Shenzhen, China.; ^3^ Department of Mechanical Engineering, City University of Hong Kong, Hong Kong, China.; ^4^ Center for Advanced Structural Materials, City University of Hong Kong Shenzhen Research Institute, Greater Bay Joint Division, Shenyang National Laboratory for Materials Science, Shenzhen, China.; ^5^ Institute for Carbon Neutrality, University of Science and Technology Beijing, Beijing, China.; ^6^ COMAC Shanghai Aircraft Manufacturing Co. Ltd., Shanghai, China.; ^7^ Jiangxi Baohang Advanced Materials Co. Ltd., Nanchang 330224, China.

## Abstract

Additively manufactured mechanical metamaterials exhibit extraordinary physical and mechanical performance. However, achieving a balance between lightweight design, strength, and energy absorption remains challenging. Here, we develop a material-structure-function integrated strategy to additively manufacture lightweight metamaterials. Specifically, strong yet ductile aluminum (Al) alloy with heterogeneous grain was developed to print hero shrew–inspired damage-resistant metamaterials. The synergistic interplay between microscale strengthening and mesoscale architectural stress regulation leads to a cross-scale coordination mechanism, which effectively bridges material heterogeneities and structural hierarchy for multilevel energy dissipation. Such a strategy enables our metamaterials to maintain a stable stress platform during deformation. Hence, our metamaterials display an excellent combination of ultralightweight (0.91 ± 0.01 g/cm^3^), high relative yield strength (17.0 ± 0.7%), and unprecedented specific energy absorption (39.1 ± 0.7 J/g), surpassing most metallic metamaterials. This facile concept expands the design space for lightweight metamaterials and demonstrates scalable strategies to realize the cross-scale coordination mechanism required by multifunction, showing transformative potential in mass production for sustainable engineering solutions.

## Teaser

A material-structural-functional route enables 3D-printed metamaterials for high-energy absorption.

## INTRODUCTION

High-performance metallic components are foundational to modern industries, indispensable for advanced engineering applications in aviation, aerospace, transportation, and energy production (*1*). In general, these components feature intricate structures, incorporating a diverse array of attributes like lightweight, superior bearing capability and good reliability (*2*). Optimizing their performance typically involves meticulous microstructural tuning at nano- and microscales through alloy composition design (*3*, *4*), controlled forming process (*5*, *6*), or precise postprocessing techniques (*7*, *8*). Structural optimization, conducted at larger scales further refines performance via shape (*9*) and size-based topology optimization (*10*–*12*). Yet, integrating these material and structural optimizations to unlock synergistic enhancements remains a formidable challenge.

Mechanical metamaterials, also known as lattice metamaterials, are engineered structures that can be flexibly designed to exhibit exceptional properties, including high strength (*13*), stiffness (*14*), toughness, super-elasticity (*15*), and energy absorption (*16*, *17*) from microscale to macroscale (*18*–*20*). The advent of metal additive manufacturing (AM; or 3D printing) has remarkably advanced the development of these materials, offering unique design freedom for complex geometries. AM’s layer-by-layer deposition manner enables fine control over the microstructure and mechanical properties of metamaterials, facilitating the near-net-shape forming of lightweight components with integrated material-structure-function properties. Recent efforts to enhance the mechanical performance of metamaterials have yielded substantial improvements in properties such as compressive strength and energy absorption. Advances include the use of stronger alloys through tuning the chemical composition (*21*) and adjusting the building direction during AM. These approaches indirectly influence the microstructure, leading to the property improvement of metamaterials (*22*). Furthermore, inspired by the nacre from mollusk shells (*23*), interpenetrating metamaterials with soft and hard matrix materials [metal-metal (*24*) and metal-polymer (*25*)] have been proposed to increase both strength and toughness. Furthermore, the performance can also be improved through structural optimizations, using biomimetic designs (*16*, *26*–*29*), gradient structures (*30*), and nonperiodic strategies (*28*, *31*–*33*).

Despite these innovations, achieving collaborative integration of material, structure, and process remains challenging. The performance of these metamaterials is typically the result of separately optimized “material” or “structure” components. As such, the material-structure-process approach often requires iterative trial and error to fine-tune the parameters in each domain to meet performance expectations. This challenge limits the breakthrough of performance and broader practical uses of metamaterials, as concluded in text S1 and fig. S1. To overcome these limitations, we propose a collaborative Innovation route that integrates lightweight materials with superior mechanical properties and optimized structural designs, offering a potential breakthrough in the performance of metamaterials. Here, we demonstrate a one-step AM approach to fabricate high-performance metamaterials by integrating material-structure-function principles. We use a tailored strong yet ductile aluminum (Al) alloy with a heterogeneous microstructure for AM, to precisely print bio-inspired damage-resistant (*34*–*36*) metamaterials with a high degree of freedom in design parameters.

**Fig. 1.**
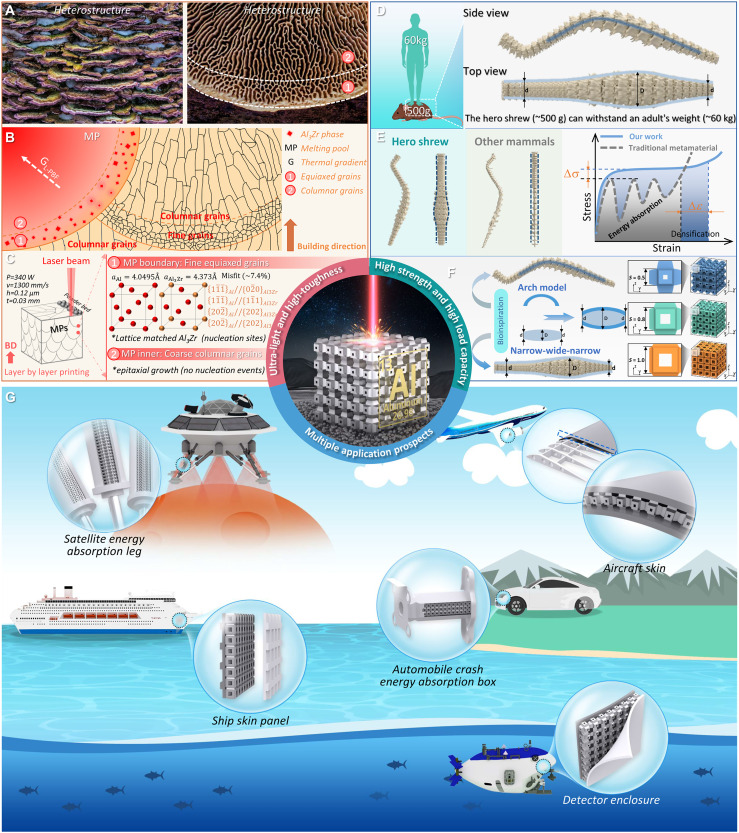
Material-structure-function integrated design strategy of bio-inspired high-performance metamaterials. (**A**) A coral fungus with a heterogeneous structure. (**B**) The hierarchically heterogeneous microstructure features a bimodal grain size distribution in MPs. (**C**) Theoretical calculation for the L-PBF of fine-grained and crack-free Al-_1.25_Mg-_0.67_Si-_0.3_Cu-_0.3_Cr-_0.2_Fe-_0.96_Zr alloy (in wt %). (**D**) Hero shrew with excellent load-bearing performance and spine characteristics. (**E**) Sketch maps showing the spine characteristics of hero shrew and other mammals, as well as the compressive curves of mechanical metamaterials inspired by them. (**F**) The structural design strategy of biomimetic metamaterials inspired by hero shrew. (**G**) The application of biomimetic metamaterials in the deep sea (detector enclosure), sea surface (ship skin panel), land (automobile crash energy absorption box), aviation (aircraft skin), and aerospace (satellite energy absorption leg). BD, building direction.

This work uses laser powder bed fusion (L-PBF) (*37*, *38*) process to print Al-based metamaterials, addressing critical requirements through synergistic material-process codesign. We develop crack-free, cost-effective Al alloys excluding precious/rare earth elements, leveraging heterostructure design principles (*39*, *40*). Bioinspired by coral fungi’s molten pool (MP)–like structure with boundary equiaxial grains and inner columnar grains (Fig. 1A), we engineered a hierarchically heterogeneous microstructure (Fig. 1B) for Al alloy. The Al-Mg-Si-Cu base alloy (similar to 6xxx series) is selected for its strength-ductility potential (*3*, *4*), with Zr addition to eliminate L-PBF hot cracks and induce bimodal grain distribution via uneven precipitation of the L1_2_-ordered Al_3_Zr nanoparticles. These lattice-matched nanoparticles promote heterogeneous nucleation at MP boundaries (Fig. 1C), while epitaxial growth along the temperature gradient (Fig. 1B) yields columnar grains. High-performance gas-atomized powders (fig. S2) combined with tailored printing/heat treatment processes produce bulk Al alloys with low density (~2.7 g/cm^3^), high tensile strength (360 to 370 MPa), and 17 to 18% elongation (fig. S3). The alloy’s stable deformation behavior (suppressed stress fluctuations) and energy absorption capacity originate from coordinated microscale strengthening and mesoscale architectural stress redistribution, establishing fundamental prerequisites for metamaterial functionality.

To endow the metamaterials with excellent mechanical properties, especially energy absorption, structures with high load-bearing capability tailored for our Al alloy are of vital importance as well. Here, inspired by the hero shrew, an amazing diminutive mammal, we propose an effective design strategy. The hero shrew (~500 g) has even been reported to withstand the weight of a full-grown man (over 60 kg) in its back (*34*, *41*). That is roughly the equivalent of a human supporting the weight of the space shuttle (*34*). Such extreme loading condition is highly related to the distinctive broad spine of the hero shrew (Fig. 1D), although its underlying mechanisms and biological evolution processes have not yet been well explained. Unlike traditional mammal-inspired metamaterials (i.e., simple cubic metamaterials), a widely reported benchmark configuration in metamaterial research (*14*, *42*), which is recognized for high compressive strength due to its orthogonal plate-like units that disperse stress effectively across the loading direction, the hero shrew’s unique spinal architecture, featuring a uniquely arched morphology in lateral view and remarkably compact dimensions in axial view (Fig. 1E), inspired our design of mechanically enhanced metamaterials. We thus adopted two critical biomimetic features: (i) the characteristic spinal curvature and (ii) longitudinal narrow-wide-narrow distribution pattern. Through the parametric control of shape factor (*S*) and thickness factor (*T*) (Fig. 1F), we achieved tunable porosity and feature size, enabling programmable mechanical performance optimization (design details in figs. S4 and S5 and text S2). This bio-inspired strategy notably expands metamaterial design freedom while maintaining structural integrity.

The material-structure-integrated metamaterials demonstrate unprecedented performance: ultralightweight (0.91 ± 0.01 g/cm^3^), high relative yield strength (17.0 ± 0.7%), and record specific energy absorption (39.1 ± 0.7 J/g), outperforming conventional metallic counterparts. This presents a systematic solution to grapple with the existing challenges in AM by cross-scale coordination. The potential applications span several critical domains (Fig. 1G), such as (i) space exploration, enhanced damping capabilities improve planetary landing safety while reducing fuel consumption; (ii) aerospace engineering, lightweight components decrease carbon emissions while maintaining maneuverability through optimized mass distribution; and (iii) automotive safety, crash energy absorption boxes that can effectively mitigate collision forces. Bio-inspired architecture also enables tunable mechanical response through parametric control allowing performance customization for multifunctional requirements. Therefore, this framework establishes a universal blueprint for developing advanced metamaterials that synergistically combine lightweight design, energy absorption efficiency, and structural durability, with demonstrated scalability for industrial production.

## RESULTS

### Multiscale characterizations of metamaterials

**Fig. 2.**
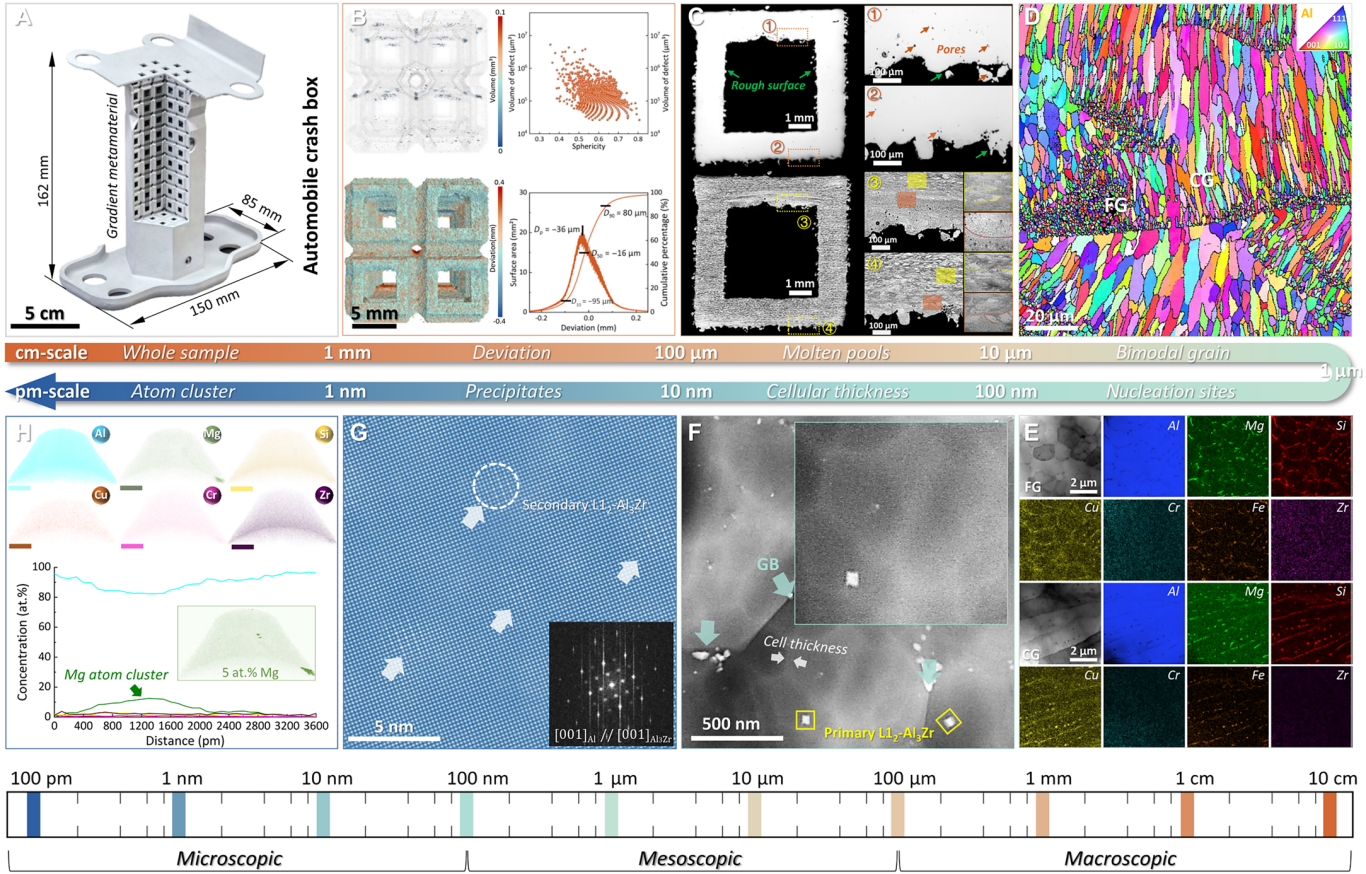
Multiscale characterization of biomimetic metamaterials from centimeters to picometers. (**A**) 3D printing of automobile crash energy absorption box with internal gradient biomimetic metamaterial. (**B**) 3D reconstruction CT images exhibiting the internal defects and surface deviations. (**C**) Optical images of the biomimetic metamaterial displaying the peacock-tail-like MPa and printing quality. (**D**) Longitudinal EBSD inverse pole figure showing the bimodal grain distribution. (**E**) TEM images and corresponding EDS mapping of the FG region and CG region revealing the uneven distribution of the L1_2_-ordered Al_3_Zr nanoparticles. (**F**) TEM image shows the submicrometer-sized cellular structures. (**G**) High-resolution TEM image of the heat-treated metamaterial presenting the high-density secondary Al_3_Zr nanoparticles, with the inset showing the corresponding fast Fourier transform. (**H**) 3D reconstructions of APT data and the corresponding proximity histograms of the Al matrix in the biomimetic metamaterial. The scale bar in (H) is 20 nm.

On the basis of the material-structure-integrated innovative strategy, the bio-inspired design of Al-based mechanical metamaterials (biomimetic metamaterials) with distinct geometrical features has been successfully built by L-PBF (fig. S6). To further analyze the printing quality and microstructure features, multiscale characterization from centimeter to picometer scale has been conducted in a typical biomimetic metamaterials model with 65% porosity and 1.0 shape factor. Figure 2A illustrates the 1:1 printing of an automobile crash energy absorption box (150 × 85 × 162 mm^3^) incorporating an internal gradient lattice metamaterial, underscoring the substantial promise of our bio-inspired strategy for advanced industrial applications. Images of the energy absorption box from other perspectives are shown in fig. S7. The micro–computed tomography (CT) results indicate that the 3D-printed biomimetic metamaterial was nearly fully dense with a high relative density of up to 99.97% while most of the internal defects (fine pores) were considered gas holes due to their high sphericity (Fig. 2B). Moreover, the deviation maps derived from the micro-CT confirmed the high printing precision with an average deviation of −36 μm. Compared with the originally designed size, the negative deviation in the printed samples is the main source of deviation, which is caused by the specific solidification shrinkage of Al alloy. In contrast, the balling phenomenon (*5*) and attached powders on the sample surface induced by the powder splashing during L-PBF led to a positive deviation (figs. S8 and S9). This high relative density value and slight printing deviation were also observed in the other biomimetic metamaterials (figs. S10 and S12 and text S3), displaying the excellent compatibility of our design strategy and printability. The optical cross-section images of the biomimetic metamaterial manifest the peacock-tail-like MPs with a width of ~200 μm and a depth of ~100 μm, indicating the high-quality interlayer bonding (Fig. 2C) (*5*).

The longitudinal electron back-scattered diffractometer (EBSD) maps of the 3D-printed biomimetic metamaterial that cover several MPs are presented in Fig. 2D and fig. S13. The hierarchical heterostructure with bimodal grain distribution consists of the submicromillimeter-sized fine grains (FGs) along the MP boundaries and the columnar grains (CGs) with a width of several micrometers and a length of ~5 to 30 μm inside the MPs. Further transmission electron microscope–energy-dispersive spectroscopy (TEM-EDS) mappings (Fig. 2E) indicate that the uneven distribution of the L1_2_-ordered Al_3_Zr nanoparticles, which have a coherent interface with the Al matrix to induce the heterogeneous nucleation under fast cooling (fig. S14), is the cause of this heterogeneous microstructure, consisting with the original intention of our alloy design strategy. Characterization down to the nanometer scale reveals the unusual cell structures with an average cellular thickness of ~50 nm, as presented in Fig. 2F. Such architecture that was hardly triggered in other metallurgical processes has been widely verified to enhance the mechanical performance in L-PBFed alloys (*43*). After a direct aging process, the bimodal grain distribution remained (fig. S15) while there were high-density secondary Al_3_Zr nanoprecipitates with a diameter of ~3.4 nm in the Al matrix (Fig. 2G). The 3D atom probe tomography (APT) maps and the corresponding chemical component analysis in Fig. 2H manifest the slight chemical fluctuation (Mg element) in the 1 to 2 nm range. These atom clusters are known to contribute to the high yield strength as they can strongly pin the mobile dislocations during the early stage of the deformation/strain process, as verified in our recent work (*4*).

### Mechanical behavior and deformation mechanisms

**Fig. 3.**
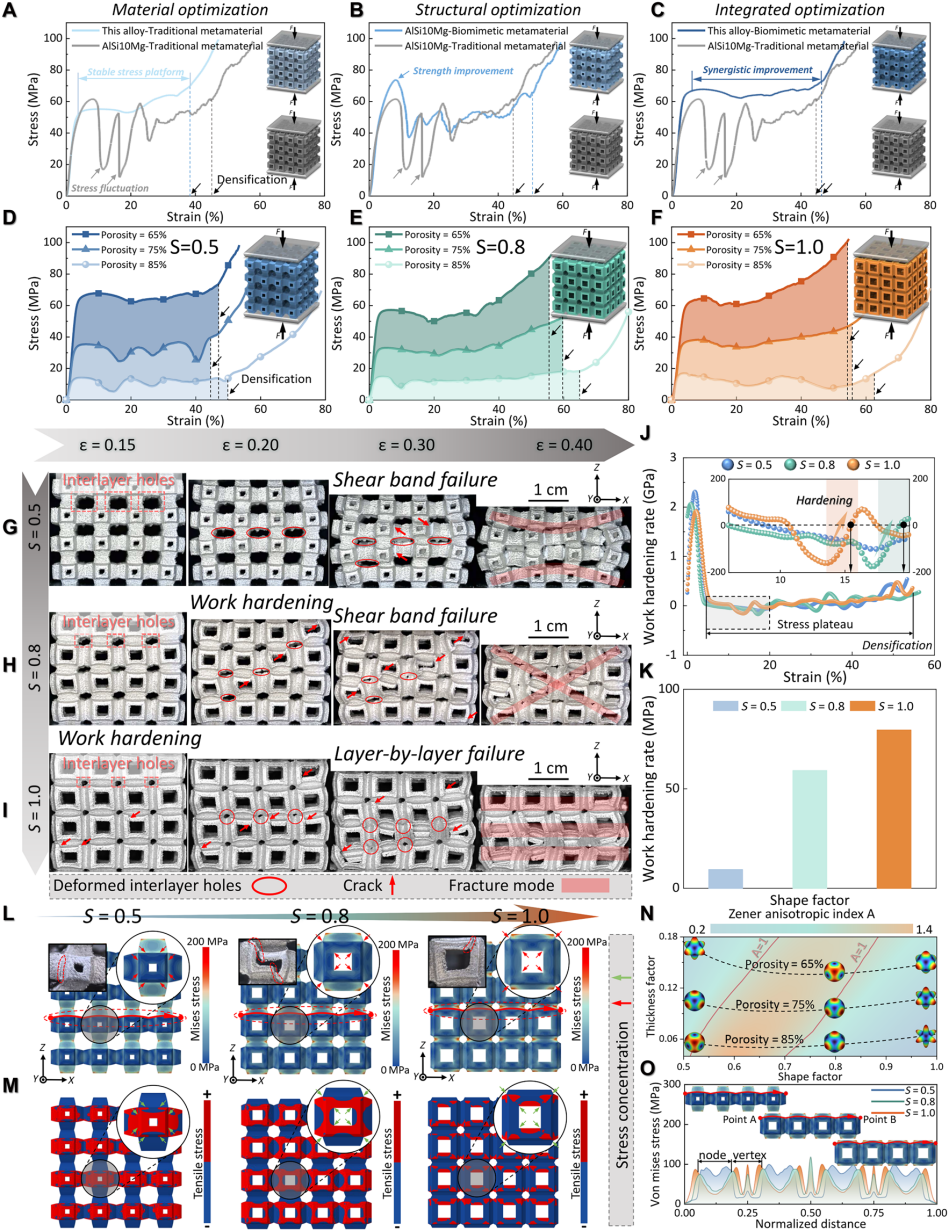
Compressive tests and finite element simulation of metamaterials with 65% porosity. Comparison of compressive stress-strain curves between traditional metamaterials fabricated with AlSi_10_Mg and metamaterials obtained via (**A**) Material optimization, (**B**) Structural optimization, and (**C**) material-structure integrated optimization. The typical compressive stress-strain curves with (**D**) 0.5, (**E**) 0.8, and (**F**) 1.0 shape factor *S*. In situ compressed images with (**G**) 0.5, (**H**) 0.8, and (**I**) 1.0 shape factor *S*, and (**J**) their work hardening rates. (**K**) Average work hardening rate for 0.5, 0.8, and 1.0 shape factor *S*. (**L**) Von Mises stress and (**M**) tensile Von Mises distribution of biomimetic metamaterials with different shape factor *S*. (**N**) The contour map of Zener anisotropic index A as a function of shape factor and thickness factor. (**O**) Von Mises stress-normalized distance curves along the arch path for biomimetic metamaterials with various shape factor *S*.

Figure 3A (A to C) presents the compressive stress-strain curves of metamaterials under different optimization strategies. Specifically, through material optimization by introducing an Al-_1.25_Mg-_0.67_Si-_0.3_Cu-_0.3_Cr-_0.2_Fe-_0.96_Zr alloy [in weight % (wt %)] with higher elongation, the stress plateau stability of the metamaterial is markedly improved (Fig. 3A): The stress fluctuation is reduced from 72.8 to 4.0%, while the relative yield strength is increased by 22.3% (fig. S16). Although only a slight improvement in specific energy absorption (2.8%) is achieved, it provides a stable stress plateau foundation for metamaterials prepared by subsequent optimization schemes. Similarly, through structural optimization (Fig. 3B), the relative yield strength of the metamaterial is increased by 19.7%, and the stress fluctuation is reduced from 72.8 to 49.8%, which in turn results in a 33.7% improvement in specific energy absorption compared to the “no optimization” state (fig. S16). Through material-structure integrated optimization (Fig. 3C), a synergistic enhancement that outperforms the additive effect of individual optimizations is achieved: The relative yield strength and specific energy absorption of the metamaterial are increased by 56.3 and 56.4%, respectively (fig. S16), fully demonstrating the advantages and advancement of the material-structure integrated design strategy in this study.

Figure 3 (D to F) manifests the compressive stress-strain curves of biomimetic metamaterials with different values of *S* and porosity under the material-structure integrated optimization strategy. Upon inspection, it is clearly observed that as porosity decreases, the overall stress-strain behavior of these biomimetic metamaterials remains quite consistent. The stress-strain curves reveal that the Al-based biomimetic metamaterials exhibit a relatively stable stress plateau without the formation of stress instability stage, suggesting that the material-structure configurations provide excellent energy absorption performance. As the porosity decreases, the stress plateau gradually rises while the densification strain gradually moves forward, and the length of the stress plateau zone decreases. Moreover, the strength of the biomimetic metamaterials initially decreases before increasing with an increase in *S*. This highlights the critical role of *S* as a key geometric parameter for regulating the mechanical properties and deformation modes of these materials, as discussed in detail in figs. S17 and S19, and text S4. To further investigate the underlying deformation mechanisms, we select the structures with 65% porosity to deeply discuss the deformation response and failure analysis during compression. This choice is based on their relatively stable stress response and the fact that the samples remain largely intact after deformation, allowing for a clearer interpretation of the metamaterials’ mechanical behavior.

The deformation mechanism was first analyzed using the in situ compression images (Fig. 3, G to I) and the work hardening rate (Fig. 3J). It is noted that the present work hardening rate only represents the first derivative of the compressive stress-strain curve of metamaterial, which is different from the concept of work hardening rate that uses true stress-strain curves in conventional bulk alloys. This work hardening phenomenon appearing in metamaterials, which is also referred to as a multilevel stress plateau in some studies (*42*, *44*), has been mentioned in many studies as having a notable effect on the enhancement of the mechanical properties of metamaterials, especially the energy-absorption capacity. It was observed that as *S* increases, the interlayer holes in the metamaterials become smaller. The smaller the hole, the faster it closes during compression, which exerts a substantial impact on the stress platform and even the densification strain of biomimetic metamaterials. As shown in Fig. 3 (G to I), for metamaterials with high *S* values (0.8 and 1.0), interlayer holes close when the strain is about 20 and 15%, respectively, and both are still in the stress plateau. For low *S* value (0.5), the interlayer holes are too large and close only when the densification strain (about 40%) approaches. This observation aligns with the work hardening rate curve in Fig. 3J, where the work hardening rate transitions from negative to zero at strains of 19.2% (*S* = 0.8) and 15.3% (*S* = 1.0). Further statistics demonstrate that the *S* value is positively correlated with the average work hardening rate of metamaterials, as presented in Fig. 3K. Therefore, increasing *S* would reduce the interlayer pore diameter of biomimetic metamaterials and gain early-stage work hardening, thereby improving the stress plateau of biomimetic metamaterials and achieving considerable energy absorption.

We then use finite element simulations to understand the closure of interlayer holes and the mechanical properties of the biomimetic metamaterials (Fig. 3, L to O). Figure S18 presents the 3D distribution of Young’s modulus (*10*, *45*). As *S* increases, the direction of maximum Young’s modulus in the biomimetic metamaterial shifts from ⟨001⟩ to ⟨111⟩, and then back to ⟨001⟩. This behavior suggests that the Zener anisotropic index (index A) of the biomimetic metamaterial can be well tuned by adjusting *S*. In other studies, the index A and size parameters mostly exhibit monotonic patterns, and this nonlinear correlation phenomenon has not been observed (*10*, *42*, *45*). To understand this unique property in our biomimetic metamaterials more clearly, Fig. 3N shows the plot of the index A for the biomimetic metamaterial. The effect of *T* on the index A follows a similar trend to that of *S*. Specifically, as *T* increases, the index A first rises and then falls, indicating that isotropic biomimetic metamaterials with various shapes can be achieved by adjusting either *S* or *T*. Figure 3 (L and M) exhibits the Von Mises stress distribution and tensile stress distribution of the biomimetic metamaterials, respectively. The magnified images in Fig. 3L highlight the unit-cell Von Mises stress distribution for each structure. For quantitative analysis, Von Mises stress values along the *x* direction (from point A to point B) were extracted from the biomimetic arch structure, and the normalized stress distribution is presented in Fig. 3O. By comparing the magnified images in Fig. 3L with the normalized stress-distance curve, when *S* = 0.5, the stress in the biomimetic metamaterial is mainly concentrated at the junction between the node and the strut. As *S* increases, this stress concentration shifts toward the vertex. Together with Fig. 3M, the type of stress in these stress-concentrated regions is mainly recognized as tensile stress. In essential, the cracks arise at the locations where the tensile stresses are the highest (*46*), which is consistent with the locations where cracks appear as observed experimentally in the magnified images of Fig. 3L. Thus, it is concluded that increasing the *S* could shift the stress concentration of the unit cell of the biomimetic metamaterial from the joint of node and strut to the vertex of the unit cell. This is conducive to the closure of the interlayer holes of the biomimetic metamaterial, and the realization of further work hardening to enhance the energy absorption of the biomimetic metamaterial. It is also worth mentioning that elastic isotropy can also be achieved under various shapes by turning *T* or *S*.

**Fig. 4.**
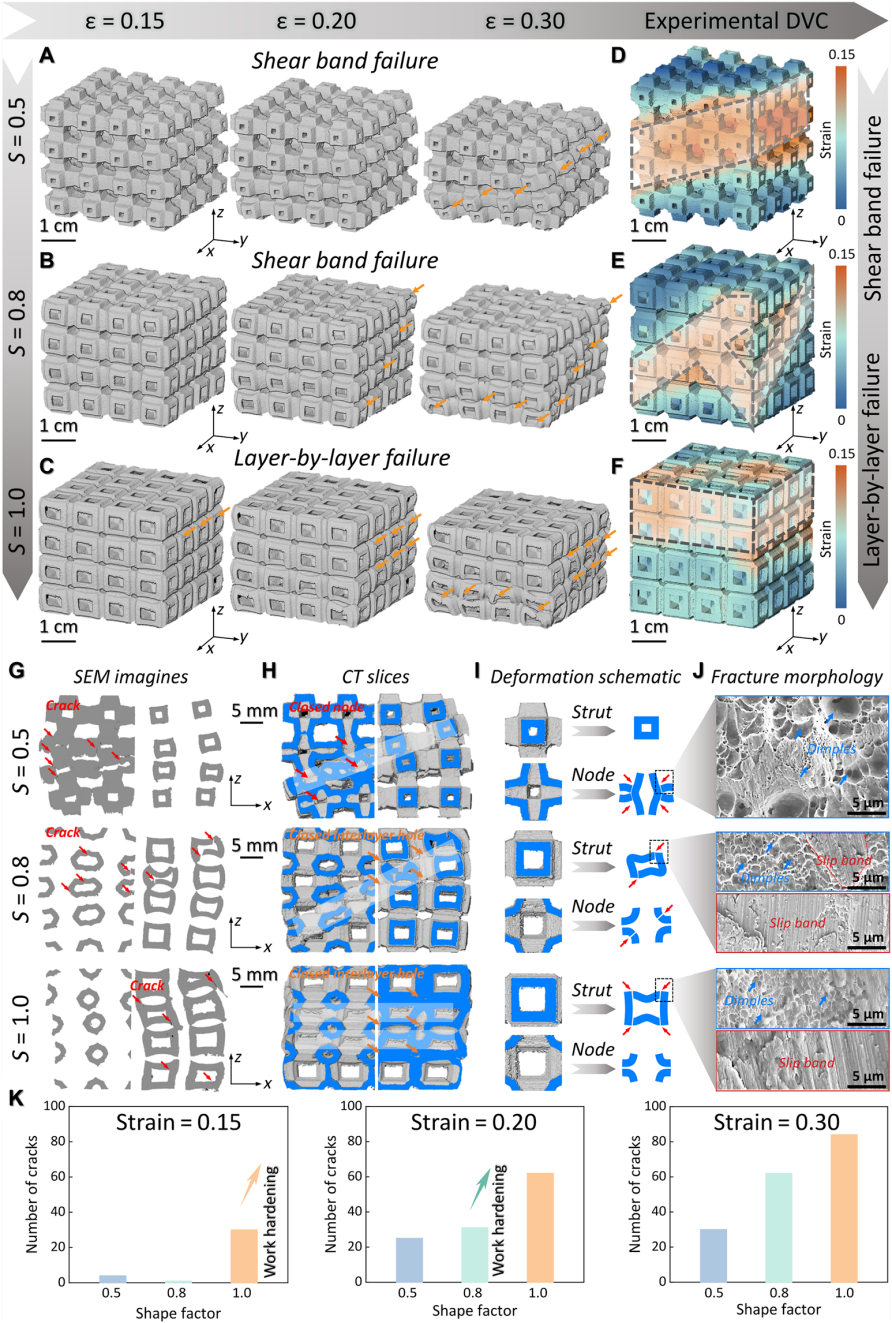
Micro-CT analysis of quasi-in-situ compression of biomimetic metamaterials with 65% porosity. The 3D reconstructed micro-CT models of biomimetic metamaterials with (**A**) 0.5, (**B**) 0.8, and (**C**) 1.0 shape factor at different strains. Digital volume correlation analysis of the reconstructed model of biomimetic metamaterials with (**D**) 0.5, (**E**) 0.8, and (**F**) 1.0 shape factor under ~0.15 strain. Characterization of biomimetic metamaterials with different shape factors: (**G**) SEM images of nodes (left) and struts (right), (**H**) micro-CT reconstructed slices of nodes (left) and struts (right), (**I**) Deformation schematics of nodes and struts, (**J**) SEM fracture surface morphology, and (**K**) crack number statistics under different strains.

Furthermore, micro-CT characterizations of the biomimetic metamaterials with 65% porosity under quasi–in situ compression were conducted to have direct visual observations to further reveal the internal deformation mechanisms, as shown in Fig. 4. Figure 4 (A to C) illustrates the 3D reconstructed models of the quasi–in situ compression experiments, with three different strain states selected for analysis given the results of the previous compression tests. The deformation mode of the biomimetic metamaterial transitions from shear band failure to layer-by-layer failure as *S* increases, which is consistent with the compressive test result in Fig. 3 (D to F). Furthermore, the strain distribution of the micro-CT–reconstructed model at 15% strain was analyzed using digital volume correlation (*47*), as depicted in Fig. 4 (D to F). For a small *S* value (*S* = 0.5), the metamaterial exhibits a shear band failure approximately two unit-cell widths wide, indicating a slightly unstable stress plateau (Fig. 3A). As the *S* increases to 0.8, the metamaterial shows a shear band failure with an “X” shape, leading to greater fluctuation in the stress plateau (Fig. 3B). When *S* increases to 1.0, the deformation mode shifts to layer-by-layer failure, which also implies enhanced energy absorption properties. The percentage of stress reduction for the three biomimetic metamaterials shown in fig. S22 quantifies the stability of the stress plateau. Because of the good toughness of the present Al alloy, the stress-strain curves of the biomimetic metamaterials did not collapse catastrophically despite the appearance of shear bands during the deformation process, which also reflects the advantage of the integrated design strategy.

In addition to the above observations, in-depth investigations into the internal deformation and fracture morphology of the biomimetic metamaterials were carried out by the sectional views presented in Fig. 4 (G to K). Initially, Fig. 4 (G to I) highlights the deformations of nodes and struts within the unit cells under a 30% strain condition. To achieve a more comprehensive understanding, the CT slices corresponding to other strain levels are presented in fig. S23. When the *S* was set at 0.5, deformation and cracks mainly occurred in the vicinity of the nodes, ultimately resulting in the closure of these nodes (Fig. 4, G and H). Meanwhile, the struts showed no obvious signs of deformation or cracking (Fig. 4I), and the interlayer holes remained open. As the *S* increased to 0.8 (Fig. 4, G and H), the cracks began to propagate through both the nodes and the struts. The nodes underwent deformation and cracking, yet they did not fully close. In contrast, the cracks in the struts led to the closure of the interlayer holes, as clearly exhibited in Fig. 4I. Subsequently, when the shape factor *S* reached 1.0, a notable shift took place: the cracks migrated toward the struts. That the interlayer holes closed at approximately 15% strain is illustrated in fig. S23. Notably, all of the fracture morphologies exhibit deeper toughness valleys (Fig. 4J). This also reflects the superior toughness of the Al alloys used in this study. Moreover, when the *S* exceeded 0.5, slip bands generated from the closure of the interlayer holes became observable. The fracture morphology at various scanning electron microscopy (SEM) magnifications is presented in fig. S24, offering a detailed microscopic perspective. Furthermore, Fig. 4K demonstrated an interesting pattern: The cracks in the struts formed earlier and were more numerous, while the node cracks formed later and were fewer in number. Therefore, increasing the value of the *S* has the effect of shifting cracks from the nodes to the struts within the unit cells, and numerous cracks in the struts markedly facilitate interlayer hole closure, regulating work hardening and enhancing the energy absorption capacity of the material.

### Integrated design-driven performance breakthrough

**Fig. 5.**
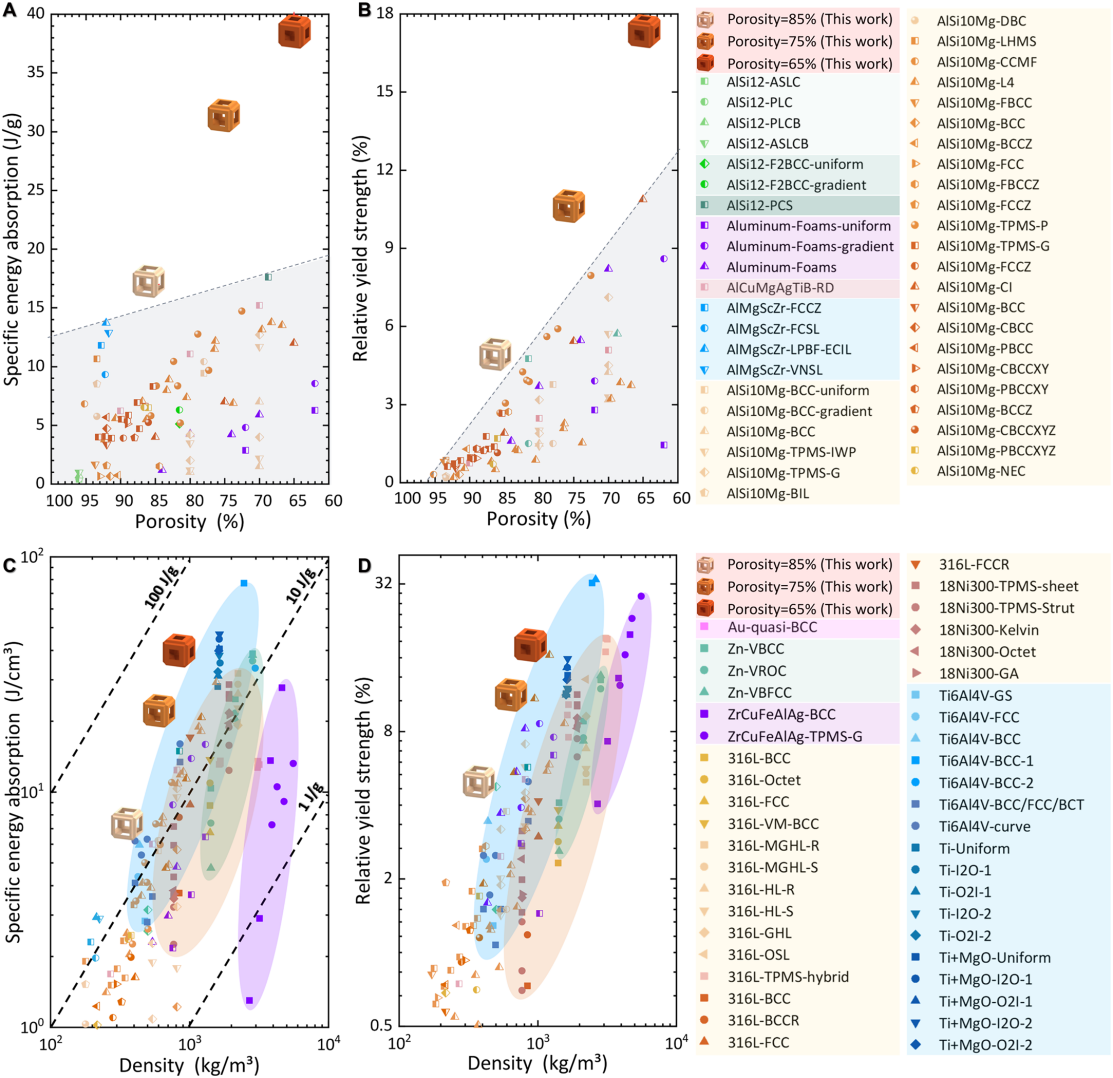
Mechanical performance of our biomimetic metamaterials. Comparison of the (**A**) Specific energy absorption and (**B**) relative yield strength of the present metamaterials with other Al-based metamaterials (*48*–*53*, *62*–*73*). Comparison of the (**C**) specific energy absorption and (**D**) relative yield strength of our metamaterials with other metamaterials made by other common alloys (*9*, *54*–*60*, *74*–*76*).

Last, we calculated the mechanical properties (relative yield strength and specific energy absorption) of our metamaterials and compared them with other metamaterials made by commonly used metallic materials, as presented in Fig. 5. Among the current Al-based mechanical metamaterials, node-reinforced FCC metamaterials printed by Al-Mg-Sc-Zr alloy (*48*) and octet metamaterials made by cast AlSi_12_ alloy (*49*) show high specific energy absorption:~near 15 and about 18 J/g, respectively (Fig. 5A). Unfortunately, the presence of notable fluctuations in the stress plateau highly limited the further enhancement of their energy absorption. For the strength, the triply periodic minimal surface (TPMS) metamaterials (*50*, *51*) and FBCC metamaterials (*52*, *53*) made from Al-Si series alloys exhibit high relative yield strength of approximately 11% (Fig. 5B). However, these Al-Si alloys often suffer from poor ductility, inevitably leading to a sharp decrease in strength after yielding. In contrast, our material-structure-function integrated design strategy successfully assembled the lightweight and high toughness of self-developed Al alloys, and the high strength by hero shrew architecture onto the biomimetic metamaterials, resulting in a high, stable, and long stress plateau during deformation (Fig. 3). This has enabled our metamaterials to achieve unprecedented mechanical performance (specific energy absorption of up to ~39.1 ± 0.7 J/g and relative yield strength of 17.0 ± 0.7%), which was substantially higher than other metamaterials with a similar porosity made by Al alloys.

In addition to the Al-based metamaterials, other metal metamaterials have also illustrated promising results, as shown in Fig. 5 (C and D). For example, TPMS metamaterials (*21*) and node-strengthened BCC metamaterials (*54*) made by Ti alloys (that are known as the alloys with the highest specific strength) also demonstrate excellent specific energy absorption (~31 J/g) and relative yield strength (~33%). Besides, mechanical metamaterials fabricated from heavier metals like Au (*55*), Zn (*56*), Zr-based metallic glass (*57*), and steels (*58*–*60*) displayed moderate specific energy absorption (often less than 17 J/g) and good relative yield strength (in some cases approaching 28%). In comparison, our metamaterials have achieved comparable or better performance than theirs in a lighter way, as indicated in Fig. 5 (C and D). This implies that we can make breakthroughs in high-performance mechanical metamaterials by material-structure-function integrated AM. Therefore, it is anticipated that the low-cost and lightweight metamaterial strategy proposed by us can be readily adopted in advanced engineering applications that require multiple functions (e.g., damping design, energy absorption, and topological optimization), as outlined in Fig. 1G.

To investigate the deformation characteristics of biomimetic metamaterials with *S* = 1.0 under different strain rates, dynamic impact tests were conducted (movie S1). Four strain rates were set in the experiments (fig. S20A): At a relatively low strain rate of 255 s^−1^, the material exhibited no obvious deformation or failure; when the strain rate increased to 672 s^−1^, deformation became gradually pronounced and concentrated at both ends, presenting a layer-by-layer failure mode; as the strain rate reached 700 s^−1^, the failure mode transitioned to shear band failure, which typically leads to a decrease in mechanical properties. The mechanical properties under different strain rates are shown in fig. S20 (B to D): as the strain rate increased from 255 to 672 s^−1^, both the yield strength and compressive strength of the material increased progressively; however, when the strain rate reached 700 s^−1^, the yield strength decreased because of the occurrence of shear band failure. Notably, the compressive strength was not affected by this failure mode, showing an increasing trend with rising strain rate and stabilizing at 65.0 MPa at 672 s^−1^. In summary, the failure mode and mechanical properties of the biomimetic metamaterial are regulated by the strain rate: Within a certain strain rate range, the deformation follows a layer-by-layer failure mode, with synchronous increases in yield strength and compressive strength; when the strain rate reaches 700 s^−1^, shear band failure causes a decrease in yield strength, while the compressive strength remains stable without a decline.

Subsequently, mechanical property comparison tests were conducted between the biomimetic metamaterial-filled crash box and a commercial crash box (6061 Al alloy), with the mass of the biomimetic metamaterial-filled crash box being 22.1% greater than that of the commercial counterpart. As shown in fig. S21A, the core energy-absorbing part of the commercial crash box was selected for the test. The quasi-static compression test results are presented in fig. S21 (B and C): The biomimetic metamaterial-filled crash box exhibited layer-by-layer failure, whereas the commercial crash box showed global buckling failure. The mechanical properties are displayed in fig. S21 (D to F), demonstrating that the biomimetic metamaterial-filled crash box achieved substantial improvements in specific energy absorption (increased by 194.7%) and yield strength (increased by 411.6%) compared to the commercial crash box.

## DISCUSSION

In summary, we have proposed a multiscale innovative design strategy to 3D print bio-inspired, lightweight, and ultrahigh-performance metamaterials through a material-structure-function integrated route. In addition, we have demonstrated that this strategy can realize collaborative innovations by assembling the high toughness of tailored lightweight Al alloys and the high strength by hero shrew architecture onto the biomimetic metamaterials, resulting in a high, stable, and long stress plateau during deformation. The present metamaterials exhibit an excellent combination of lightweight, high relative yield strength, and exceptional specific energy absorption, markedly better than that of almost all Al-based and other metallic metamaterials by AM and other routes. Our concept opens opportunities for: (i) design and printing of damage-tolerant metamaterials with desired strength and toughness, and (ii) enhancing the functionality and performance of architected materials in response to external loads in advanced engineering uses.

## MATERIALS AND METHODS

### Sample fabrication

The gas-atomized Al-_1.25_Mg-_0.67_Si-_0.3_Cu-_0.3_Cr-_0.2_Fe-_0.96_Zr powders (in wt %) were used as raw materials for the L-PBF fabrication of biomimetic metamaterials and bulk alloys. A commercial SLM Solutions 280 HL printer was used for the L-PBF process. The optimized parameters were a laser power of 360 W, a scan speed of 1200 mm/s, a hatch space of 100 μm, a layer thickness of 30 μm, and the scanning direction was alternately rotated by 67° between adjacent layers. The four-layer biomimetic metamaterials (32 mm × 32 mm × 32 mm) were used for mechanical properties testing. The cylinder sample (17 mm diameter, 76 mm height) was printed both laterally and longitudinally for tensile testing. All these samples were prepared on a 6061 Al alloy substrate under the protection of high-purity argon (99.99%). The optimized heat treatment is an aging process that was performed at 400°C for 4 hours.

### Multiscale characterization

The internal structure of biomimetic metamaterials was examined using a GE-Phoenix micro-focus CT (micro-CT) machine at a working voltage of 165 kV and a current of 120 μA. A spatial resolution of 2024 × 2024 pixels and a CT scan step size of 0.36° were adopted for rotation ranging from 0° to 360° to gain the raw data. The VGSTUDIO Max 3.2 software was then used to reconstruct the 3D data field to reveal the internal defect characteristics and overall relative density. The L-PBF samples for microstructure characterization were subjected to a standard metallographic procedure and then chemically etched using the Keller’s reagent at room temperature.

A 50-megapixel high-resolution camera was used for the macroscopic characterization of the biomimetic metamaterials. The L-PBFed metamaterials for microstructure characterization were subjected to a standard metallographic procedure and then chemically etched using the Keller’s reagent at ambient temperature. A Zeiss Axio Observer 3 optical microscope was used to capture the macrostructure features of biomimetic metamaterials. The morphology of the feedstock powders and biomimetic metamaterials were captured using a Thermo Fisher Scientific Apreo2 field-emission scanning electron microscope. An EBSD, mounted on the Apreo2 SEM, was used with a step size of 100 nm to obtain the grain size and orientation information. The detailed microstructure of biomimetic metamaterial was characterized by a FEI Talo F200X TEM equipped with EDS detectors and operated at an accelerating voltage of 200 kV. The FEI Titan Themis G2 TEM was also used at a working voltage of 200 kV to further analyze the nanoprecipitates in terms of their size and distribution behavior.

The needle-shaped specimen required for APT was fabricated by lift-outs and annular milled in an FEI Scios focused ion beam. The APT characterizations of the biomimetic metamaterials were carried out in a local electrode atom probe (CAMEACA LEAP 5000 XR). The specimen was analyzed at 70 K in voltage mode, at a pulse repetition rate of 200 kHz, a pulse fraction of 20%, and an evaporation detection rate of 0.2% atom per pulse. The data analysis workstation AP Suite 6.3 was used for creating the 3D reconstructions and data analysis.

### Mechanical property test

Dynamic mechanical property tests were conducted on an ALT1000 split Hopkinson pressure bar. Four strain rates (255, 502, 672, and 700 s^−1^) were set in this experiment. The stress and strain values were calculated by capturing reflected waves and transmitted waves, while the sample deformation was recorded using high-speed photography with 5000 frames per second. An Instron 3382 machine was used to conduct the tensile tests of the bulk samples and the compression tests of metamaterials at ambient temperature. Tensile tests were conducted along both transverse and longitudinal directions to verify isotropy using the rod tensile specimens with the gauge dimensions of 25 mm (length) × 5 mm (diameter) at a constant tensile rate of 1.5 mm/min. In the quasi-static uniaxial compression process, a high-resolution camera was used to detect the deformation of the biomimetic metamaterial, and the compression rate was kept at 1.92 mm/min. To evaluate the mechanical performance of biomimetic metamaterials, various indicators have been proposed. These include the relative yield strength (σ_lattice_/σ_solid_), the energy absorption efficiency (η), the densification strain (ε*_d_*), the specific energy absorption, and the energy absorption per unit volume (*W*) (*21*). In addition, the work hardening rate was introduced in this study as an indicator of the mechanical properties of biomimetic metamaterials during the large deformation phase. It is noted that the present work hardening rate in the present work only represents the first derivative of the compressive stress-strain curve of metamaterial, which is different from the concept of work hardening rate that uses true stress-strain curves in conventional bulk alloys. The relative yield strength (σ_lattice_/σ_solid_) is defined as (*61*)

$$\frac{\sigma_{\text{lattice}}}{\sigma_{\text{solid}}}\times 100\%$$

where σ_lattice_ represents the yield strength of metamaterial, and σ_solid_ is the yield strength of bulk Al alloys. The energy absorption efficiency (η) was obtained using the equation

$$\eta =\frac{\int_{0}^{\varepsilon_{0}}\sigma \left(\varepsilon \right)d\left(\varepsilon \right)}{\sigma \left(\varepsilon_{0}\right)}$$

where σ(ε) and ε are respectively the nominal stress and nominal strain of biomimetic metamaterial, and the parameters ε_0_ and σ(ε_0_) are the given nominal strain and the maximum nominal stress at the corresponding interval respectively. When the energy absorption efficiency (η) reached the maximal value in energy absorption efficiency-strain curve, the corresponding nominal strain was defined as densification strain (ε*_d_*), which could be governed as (*45*)

$$\frac{d\eta \left(\varepsilon \right)}{d\varepsilon }|{}_{\varepsilon =\varepsilon_{d}}=0$$

The specific energy absorption (*SEA*) and the energy absorption per unit volume (*W*) are defined by the following equations, respectively

$$\text{SEA}=\int_{0}^{\varepsilon_{d}}\sigma \left(\varepsilon \right)d\left(\varepsilon \right)$$

$$W=\left(\text{SEA}\right)\;\rho_{s}\left(\text{VF}\right)=\left(\text{SEA}\right)\;\rho_{s}\left(1-\rho_{l}\right)$$

where ρ*_s_* is the density of bulk Al alloys, ρ_*l*_ is the porosity of the biomimetic metamaterial, and volume fraction (*VF*) denotes its solid component volume fraction; the sum of VF and ρ_l_ equals 1. The work hardening rate (*WHR*) is defined as

$$\text{WHR}=\frac{d\sigma \left(\varepsilon \right)}{d\varepsilon }$$

Micro-CT analysis of quasi–in situ compression of biomimetic metamaterials was also carried out to further understand the deformation mechanisms. In particular, biomimetic metamaterials were unloaded at 0.15, 0.20, and 0.30 strains, and micro-CT analysis was immediately performed on the samples to reveal their internal structural characteristics.

### Finite element simulations

The compression process and elastic response simulations performed by the implicit solver ABAQUS/Standard were carried out to obtain the deformation behavior and Young’s modulus. The original computer-aided design models of four-layer biomimetic metamaterial were used in the compression simulations. A usual four-node tetrahedral element was used to divide the mesh. The material constitutive model considered in this study is linearly elastic without postyield behaviors. According to the tensile data, the static mechanical parameters of the elastic modulus and Poisson’s ratio of the base alloy constructed by L-PBF are 70 GPa and 0.3, respectively.

## Supplementary Materials

### The PDF file includes:

Texts S1 to S4 Figs. S1 to S24 Legend for movie S1
