# Supplementary material for: Bio-inspired material-structure-function integrated additive manufacturing of Al-based metamaterials with surpassing energy absorption
Source: Sci Adv. 2025 Nov 14;11(46):eaea0430. doi: 10.1126/sciadv.aea0430 (PMC12617464; doi:10.1126/sciadv.aea0430)
Supplement: Supplementary file 1 — Texts S1 to S4 Figs. S1 to S24 Legend for movie S1 [file sciadv.aea0430_sm.pdf]

Science Advances
AAAS

Supplementary Materials for

Bio-inspired material-structure-function integrated additive manufacturing of Al-based metamaterials with surpassing energy absorption

Xi He *et al.*

Corresponding author: Jian Lu, jianlu@cityu.edu.hk; Qiang Zhu, zhuq@sustech.edu.cn

Sci. Adv. **11**, eaea0430 (2025)
DOI: 10.1126/sciadv.aea0430

The PDF file includes:

Texts S1 to S4
Figs. S1 to S24
Legend for movie S1

Other Supplementary Material for this manuscript includes the following:

Movie S1

Supplementary Text

S1: Strategies for improving mechanical properties of metamaterials

As shown in Fig. S1, current research on mechanical metamaterials mainly uses metal materials such as 316L, Ti, or Al-Si alloys to form them. However, these matrix materials do not achieve good compatibility with mechanical metamaterials, which limits the further improvement of the mechanical properties of metamaterials. First of all, the specific strength of 316L stainless steel is relatively low, which limits the improvement of mechanical properties of mechanical metamaterials to a certain extent. At the same time, 316L also has good plasticity, and few cracks appear in the deformation process of metamaterials, which will lead to metamaterials deformation accumulation and advanced densification, reducing energy absorption performance. Second, although the density of Ti alloys and Al-Si alloys is low, their plasticity is poor, which will cause the stress platform of the metamaterial to fluctuate sharply and reduce its mechanical properties. Therefore, by designing lightweight Al alloys with good plasticity and combining them with structural design, we obtained mechanical metamaterials with excellent bearing capacity, which are expected to achieve further breakthroughs in the mechanical properties of such materials.

S2: Derivation of the porosity formula for biomimetic metamaterials

Biomimetic metamaterials can be decomposed into *Volume A* and *Volume B*. *Volume A* and *Volume B* can be decomposed into six struts and one node, the volume of which are calculated by the following formula:

$$v(S) = 6V_1 + V_2 = L^3 S^2 [3 - \frac{3S}{\sqrt{2.25 + S^2}} - 1.5^{-2}]$$

where L is unit-cell length (L=8 mm) and S is the shape factor (S).

The formula for calculating VF of *Volume A* and *Volume B* is as follows:

$$vf(S) = \frac{v(S)}{L^3} = S^2 [3 - \frac{3S}{\sqrt{2.25 + S^2}} - 1.5^{-2}]$$

The corresponding biomimetic metamaterials porosity is calculated as follows:

$$VF = vf(S) - vf(S-2T) - = S^2 [3 - \frac{3S}{\sqrt{2.25 + S^2}} - 1.5^{-2}] - (S-2T)^2 [3 - \frac{3(S-2T)}{\sqrt{2.25 + (S-2T)^2}} - 1.5^{-2}]$$

$$Porosity = 1 - VF = 1 - S^2 [3 - \frac{3S}{\sqrt{2.25 + S^2}} - 1.5^{-2}] + (S-2T)^2 [3 - \frac{3(S-2T)}{\sqrt{2.25 + (S-2T)^2}} - 1.5^{-2}]$$

where T is the thickness factor (T). The porosity of biomimetic metamaterials is mainly controlled by S and T. It goes without saying that the porosity decreases with increasing T. But as S increases, the porosity first increases and then decreases. Nine samples have been marked in Fig. S5.

S3: Surface quality of biomimetic metamaterials

Figs. S8-S9 show SEM images of the top and side surfaces of biomimetic metamaterials, respectively. From the SEM image, we can clearly see the scanning trajectory, stair effect and adhered powders phenomenon introduced by the L-PBF process, in addition, there are balling phenomena of Al alloy. Adhered powders and balling phenomenon have a great influence on the surface finish of the sample, especially on the sides of biomimetic metamaterials. In addition, it is found that the interlayer pores of the biomimetic metamaterials become smaller as the *S* increases, which will affect the deformation mode and mechanical properties of the biomimetic metamaterials.

S4: Analysis of mechanical properties of biomimetic metamaterials

Fig. S17 illustrates the comparison of mechanical properties of biomimetic metamaterials with different *S*. The pattern of variation of relative yield strength in Fig. S17A is strongly related to the Zener anisotropic index in Fig. 3L as the porosity of the metamaterial and the *S* increase. Fig. S17B demonstrates that, as the *S* increases, the metamaterial's densification strain first increases and then decreases. In this case, the cause of the first increase is the work hardening during the deformation of the metamaterial with *S*=0.8. Whereas, when *S* increases from 0.8 to 1.0, the reason that leads to a decrease in the densification strain is that the metamaterial with *S*=0.8 produces a shear band failure during deformation, and this deformation mode causes a decrease in the stacking effect of the structure, which in turn increases the densification strain. In contrast, the metamaterial with *S*=1.0 prematurely densifies due to layer-by-layer failure, which leads to structural stacking. The specific energy absorption of the metamaterials in Fig. S17C is determined by a combination of the two aforementioned reasons. At high porosity (~75%), the difference in the plateau stresses of the metamaterials is very small, and thus the main factor determining the magnitude of the specific energy absorption is the densification strain. As the porosity decreases (~65%), the difference in the densification strain of the metamaterials is very small, and at this point, the plateau stress is the main factor determining the magnitude of the specific absorption energy.

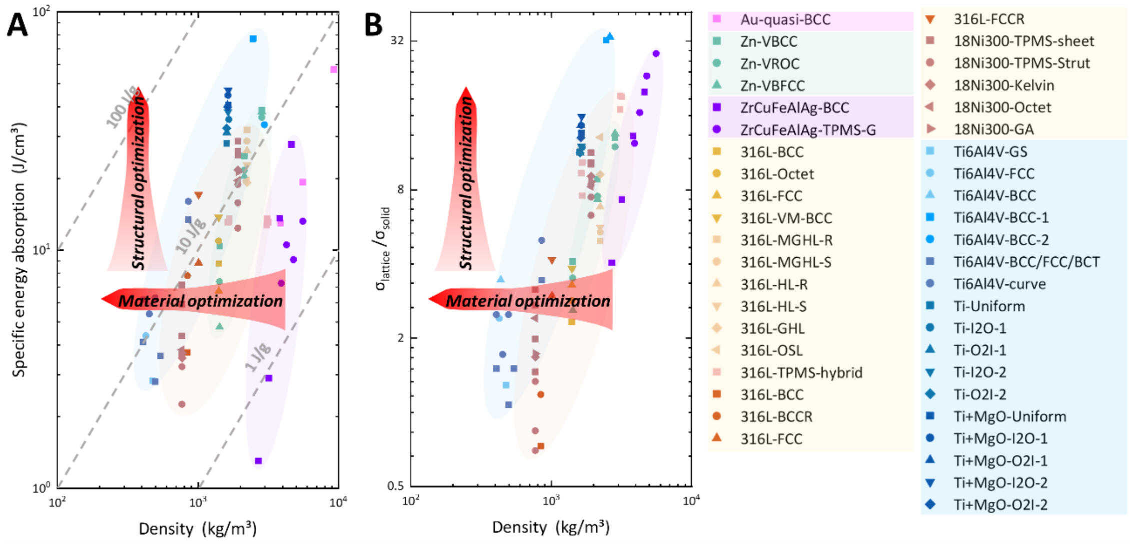

Fig. S1. Mechanical properties of metal-lattice metamaterials. (**A**) Specific energy absorption and (**B**) Relative yield strength.

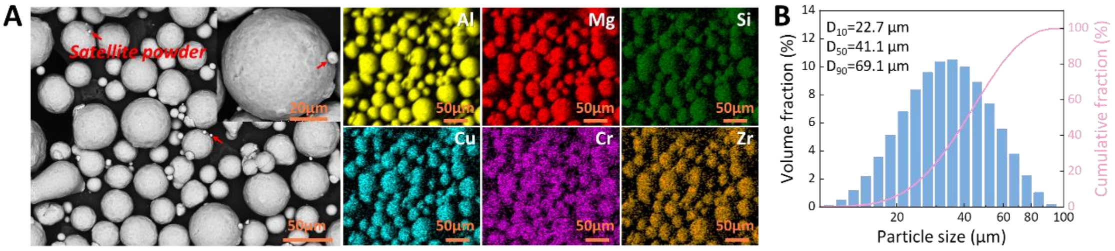

Fig. S2. Microscopic morphology and particle size analysis of the powder. (**A**) SEM and EDS mapping images of the original powder; (**B**) Particle size distribution of the powder.

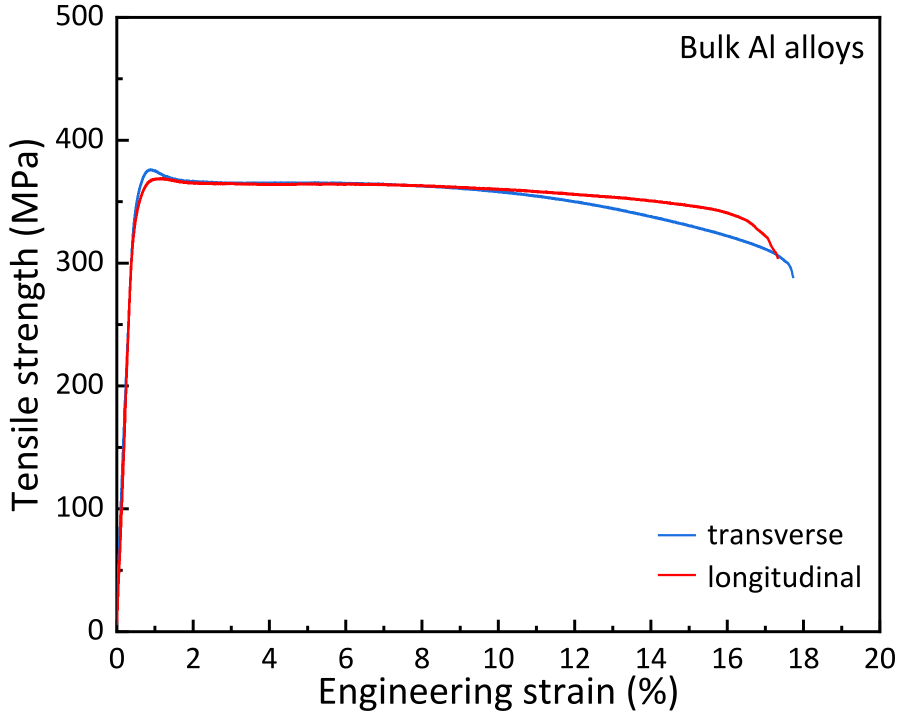

Fig. S3. Typical tensile stress curves of bulk Al alloys along both transverse and longitudinal directions.

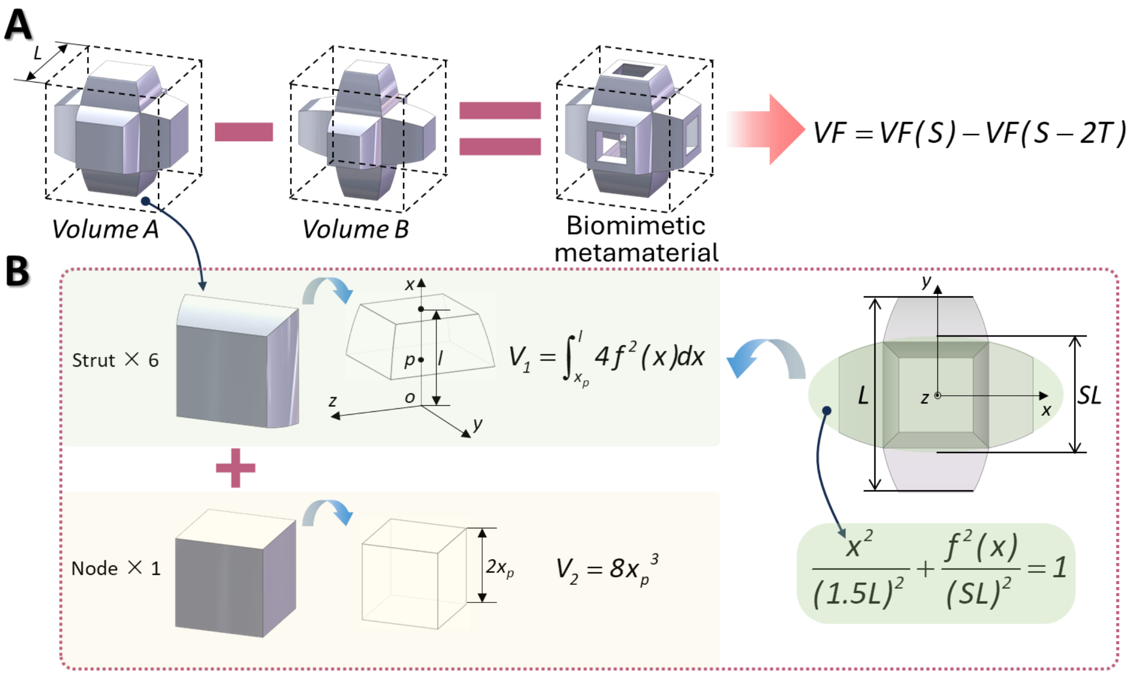

Fig. S4. Derivation of the porosity formula for biomimetic metamaterials. Structural decomposition and volume calculation of (**A**) Biomimetic metamaterials and (**B**) Volumes A and B.

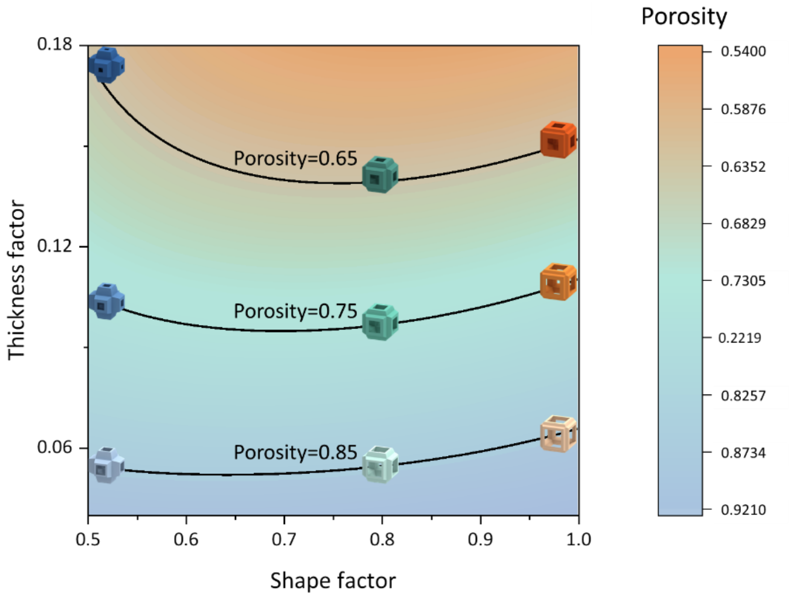

Fig. S5. The contour map of porosity as a function of *S* and *T*.

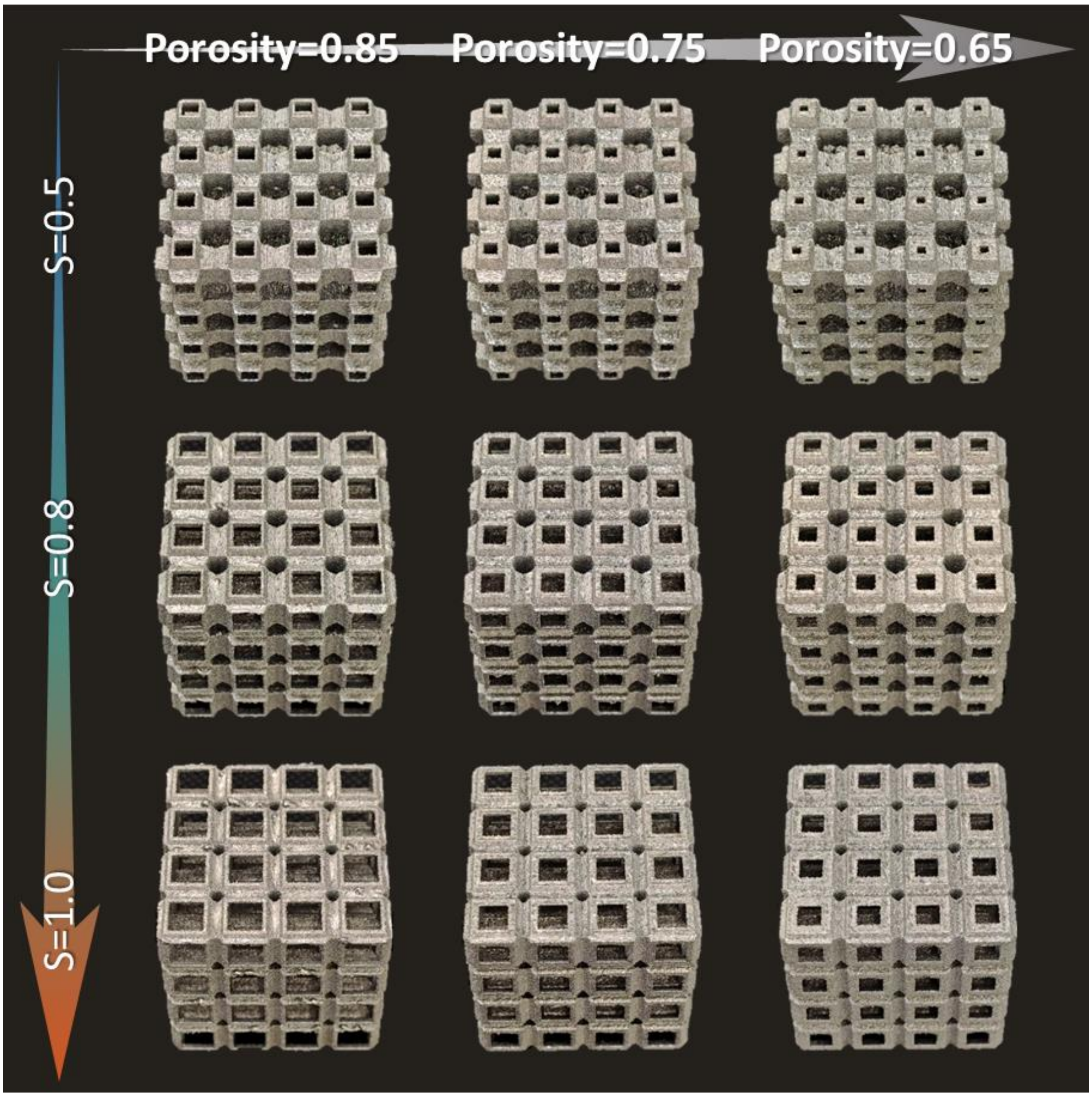

Fig. S6. The biomimetic metamaterials with different porosity and *S* fabricated via L-PBF.

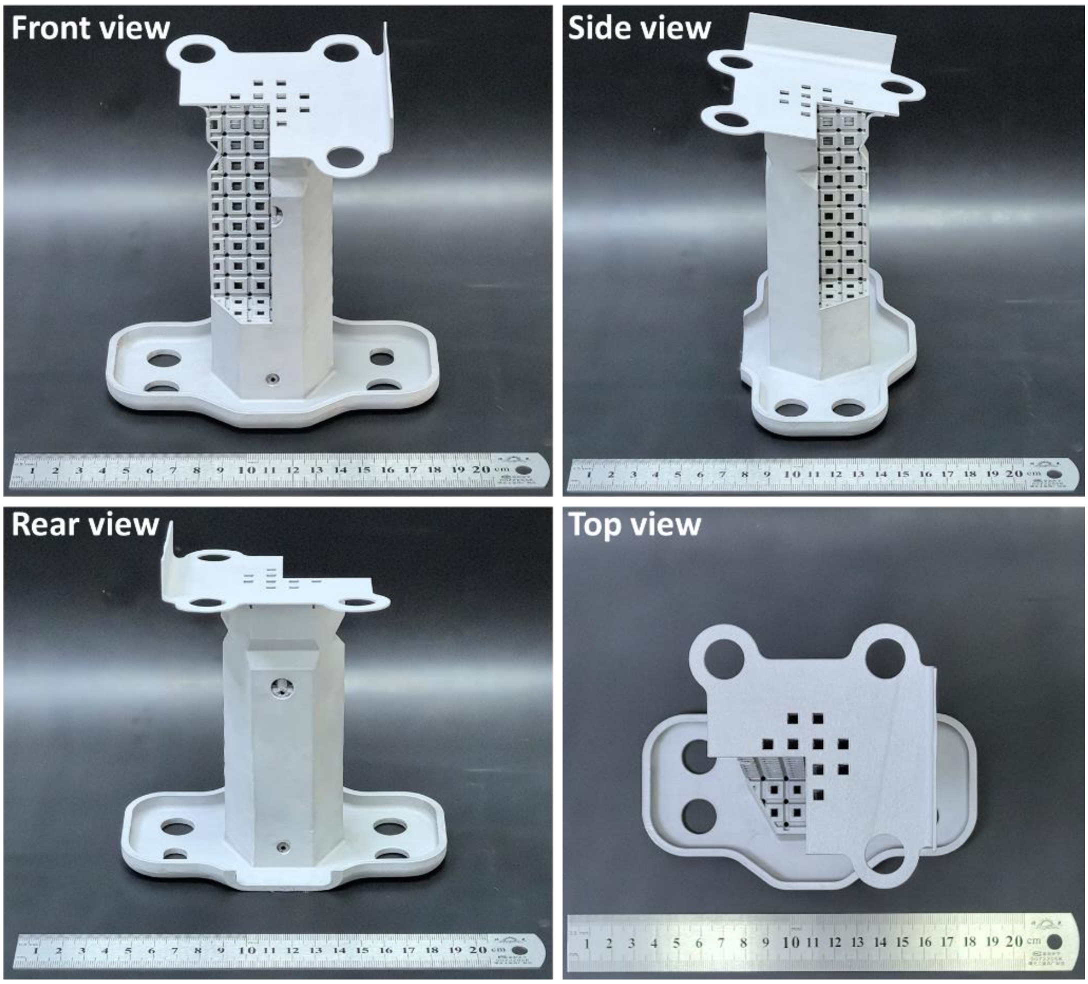

Fig. S7. Images of different perspectives of the 3D-printed automobile crash energy absorption box with internal gradient biomimetic metamaterial.

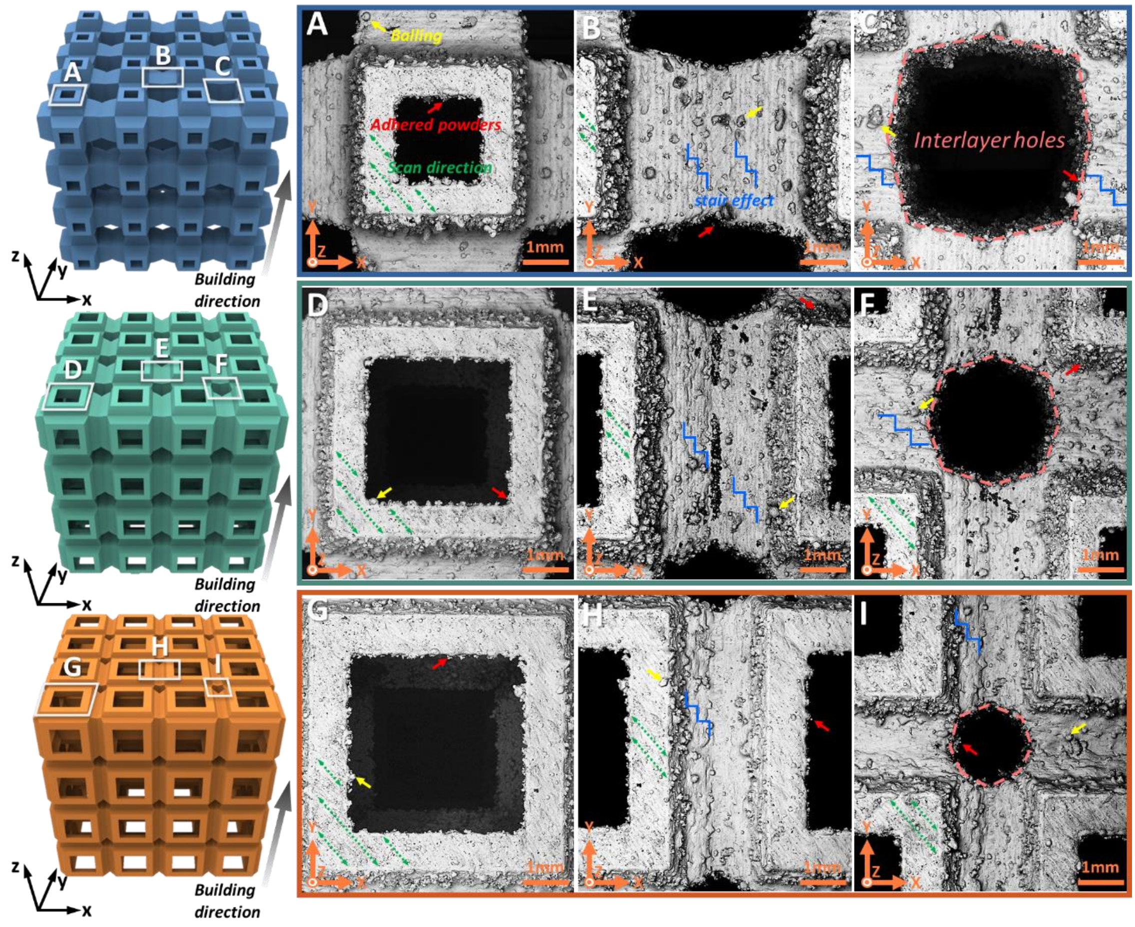

Fig. S8. Scanning electron microscopy images of the top surfaces of LPBF-fabricated biomimetic metamaterials with different *S* values. (**A** to **C**): Top surface morphologies at *S*=0.5; (**D** to **F**): Top surface morphologies at *S*=0.8; (**G** to **I**): Top surface morphologies at *S*=1.0.

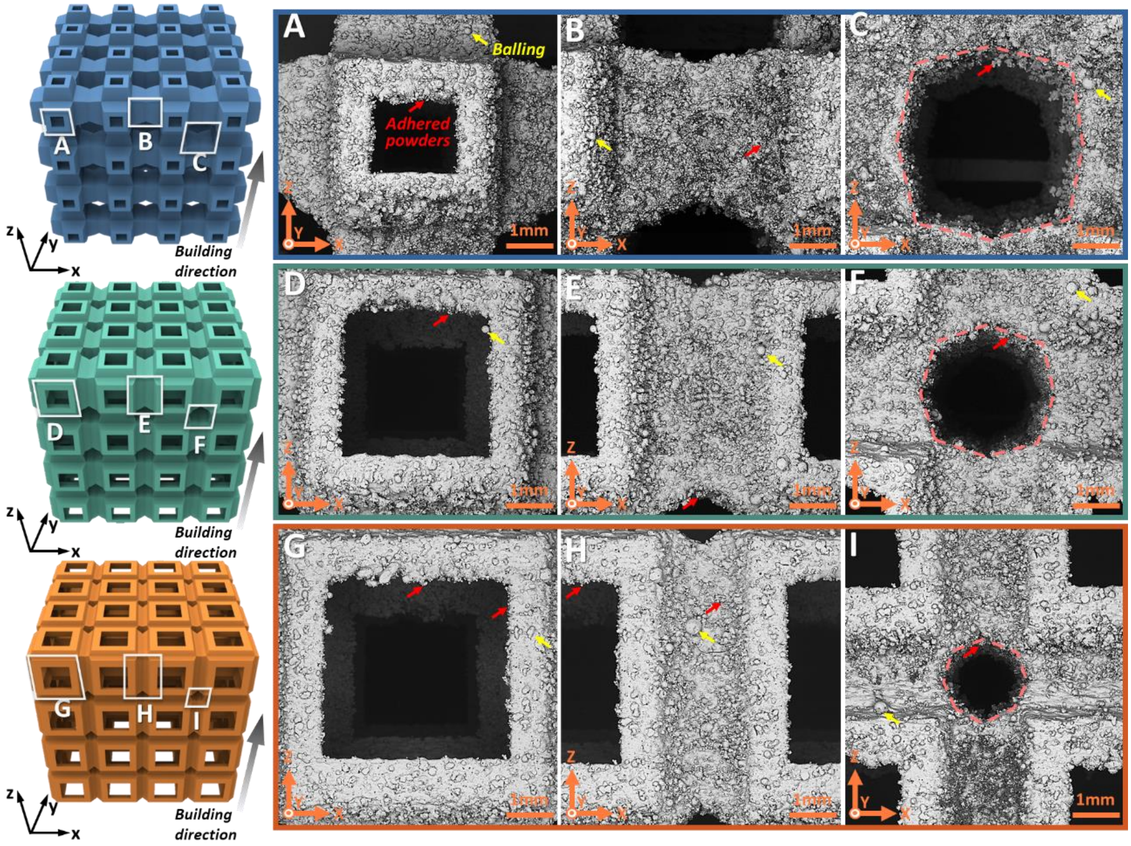

Fig. S9. Scanning electron microscopy images of the side surfaces of LPBF-fabricated biomimetic metamaterials with different *S* values. (**A** to **C**): Side surface morphologies at *S*=0.5; (**D** to **F**): Side surface morphologies at *S*=0.8; (**G** to **I**): Side surface morphologies at *S*=1.0.

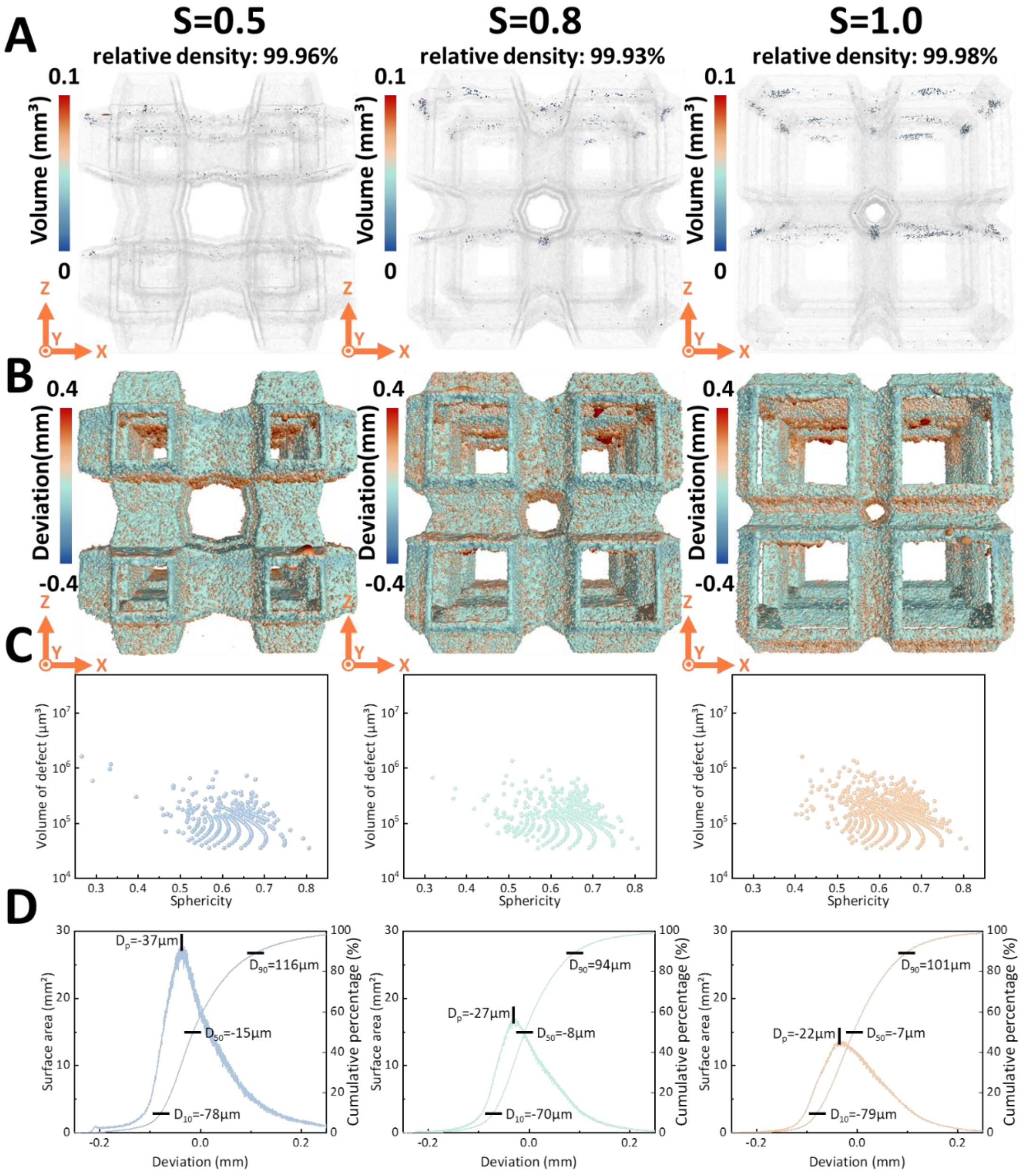

Fig. S10. Surface deviation nephogram of computer-aided design (CAD) and computed tomography (CT)-reconstructed 3D printed biomimetic metamaterial models, and void defect distribution with 85% porosity and different *S*. (**A**) The void defect distribution. (**B**) Distributions of surface deviations. (**C**) Void volume versus sphericity curves. (**D**) Surface deviation versus deviation curves.

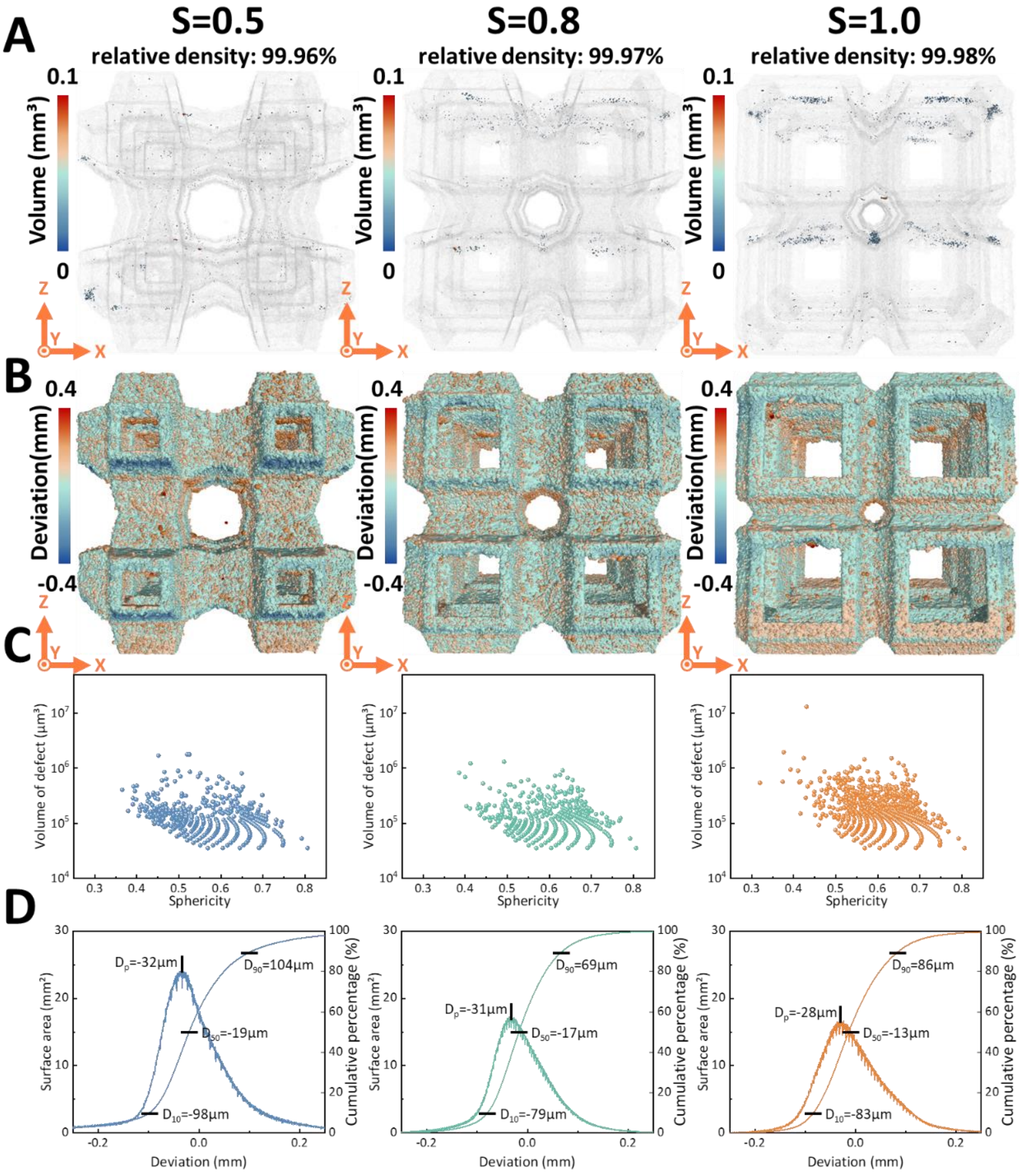

Fig. S11. Surface deviation nephogram of CAD and CT-reconstructed 3D printed biomimetic metamaterial models, and void defect distribution with 75% porosity and different *S*. (**A**) The void defect distribution. (**B**) Distributions of surface deviations. (**C**) Void volume versus sphericity curves. (**D**) Surface deviation versus deviation curves.

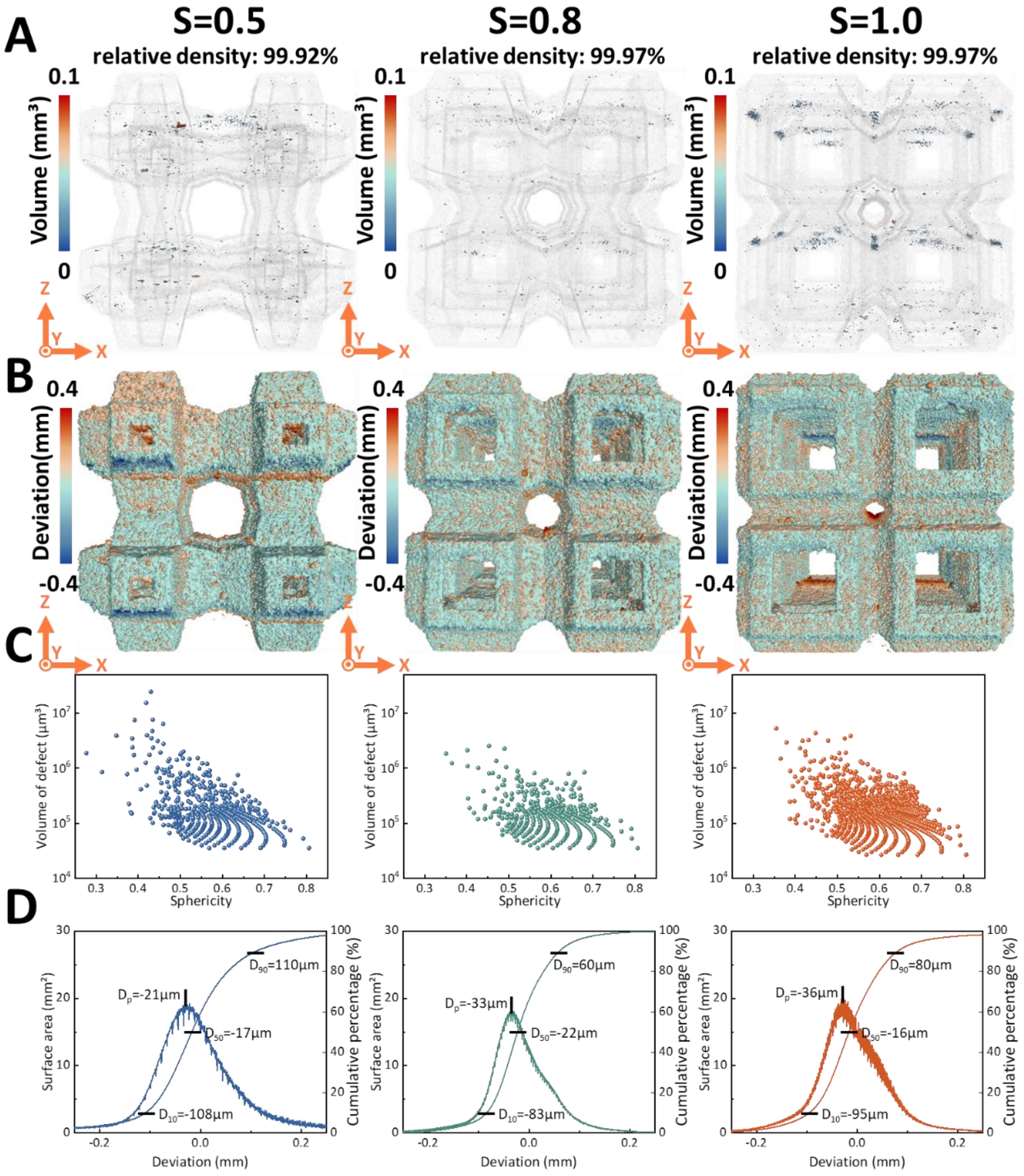

Fig. S12. Surface deviation nephogram of CAD and CT-reconstructed 3D printed biomimetic metamaterial models, and void defect distribution with 65% porosity and different *S*. (**A**) The void defect distribution. (**B**) Distributions of surface deviations. (**C**) Void volume versus sphericity curves. (**D**) Surface deviation versus deviation curves.

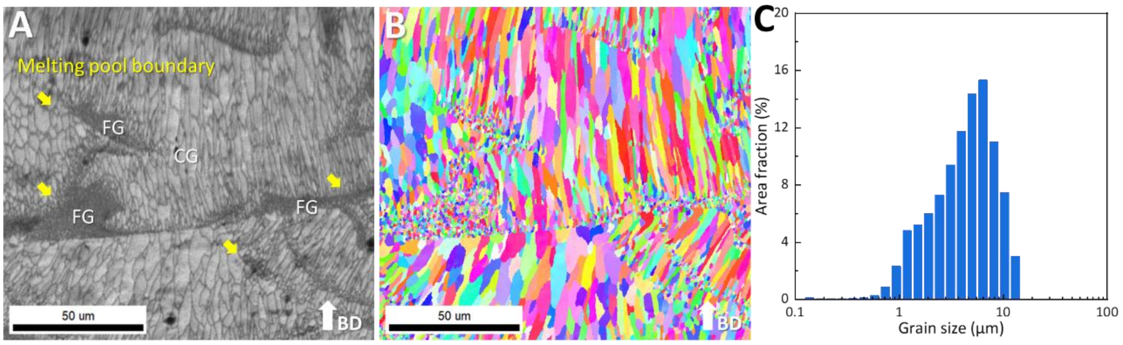

Fig. S13. Longitudinal EBSD maps of as-printed Al-based metamaterials. (**A**) EBSD image quality (IQ) map showing the FG zones and CG zones. (**B**) EBSD-IPF color image without highlighting the grain boundaries. (**C**) The grain size distribution collected from IPF map.

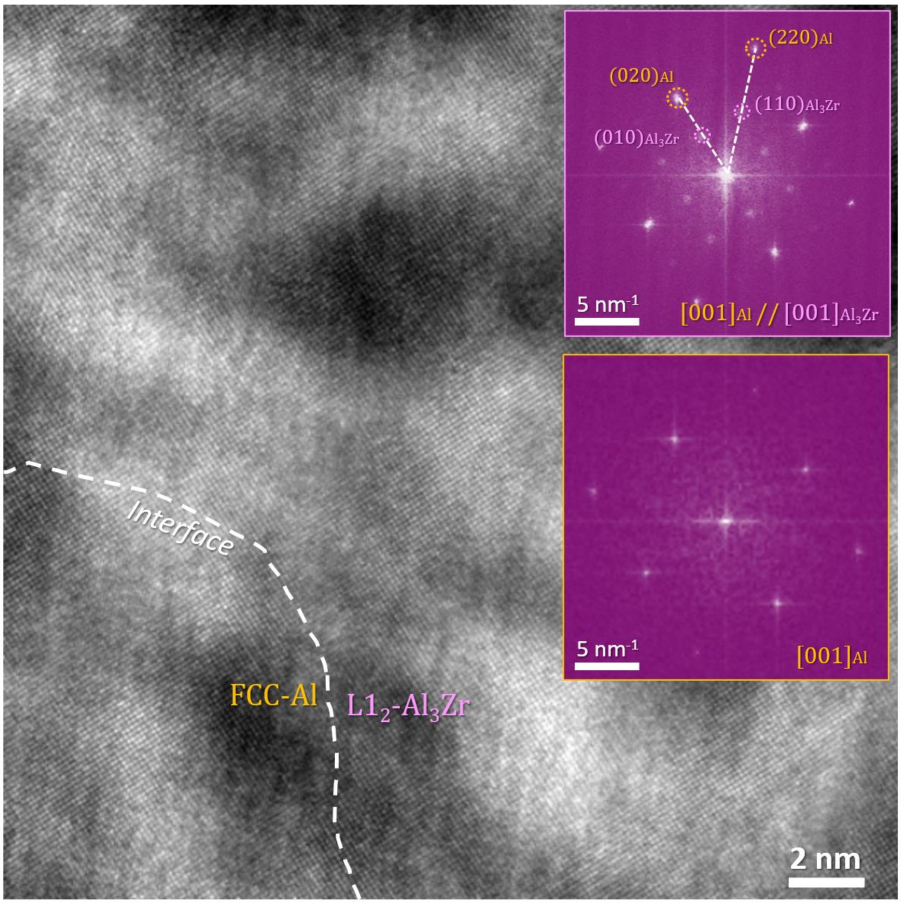

Fig. S14. HAADF-STEM image showing the microstructure of the $L1_2$-Al_3Zr/FCC-Al interface. The insets show the corresponding fast Fourier transform.

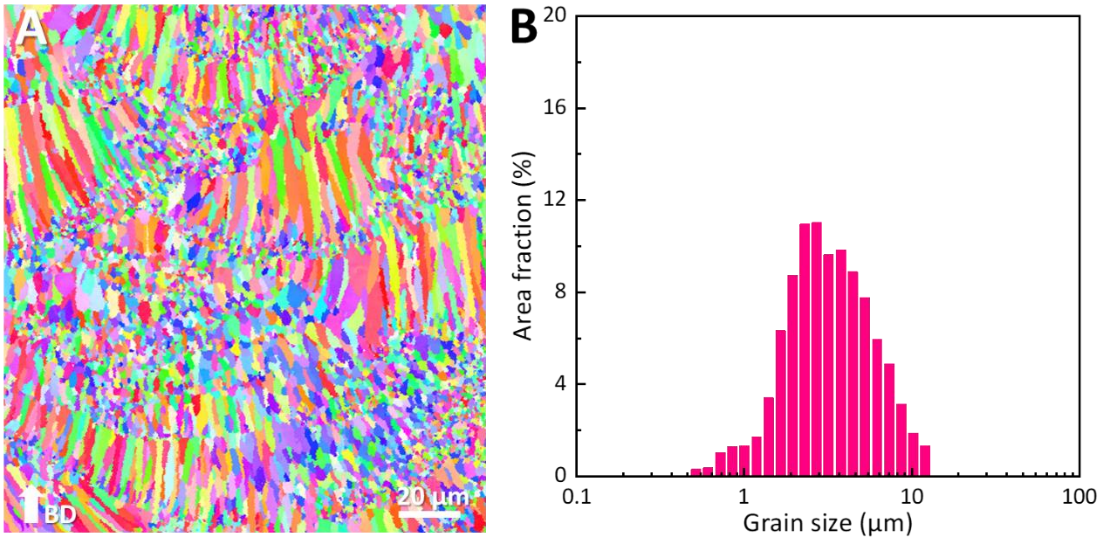

Fig. S15. Longitudinal EBSD maps of heat-treated Al-based metamaterials. (**A**) EBSD-IPF color image without highlighting the grain boundaries. (**B**) The grain size distribution collected from IPF map.

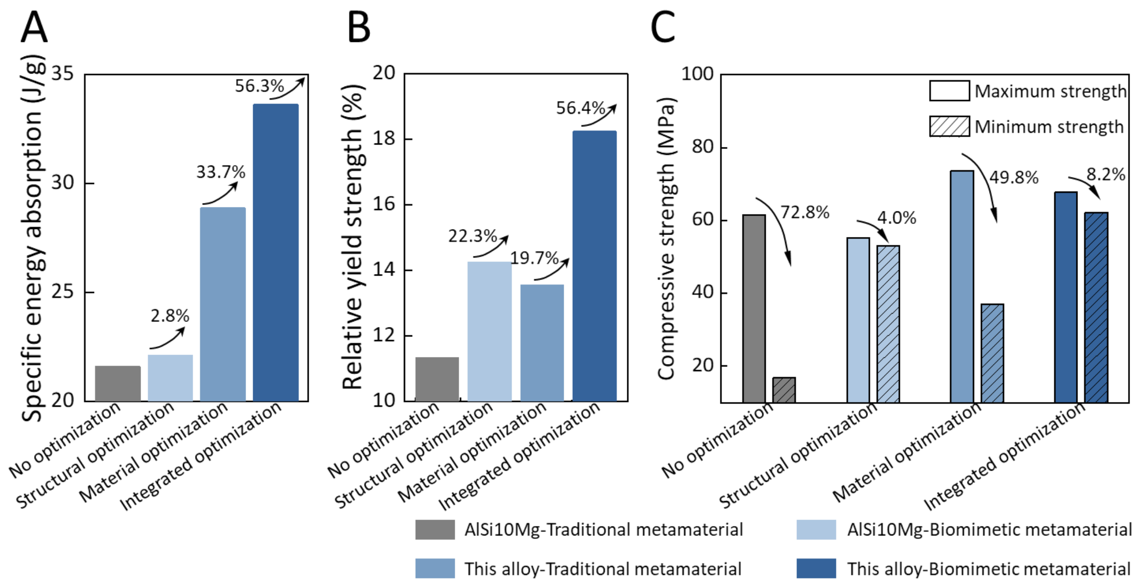

Fig. S16. Mechanical properties of metamaterials under different optimization strategies. (**A**) Specific energy absorption; (**B**) Relative yield strength; (**C**) Compressive strength.

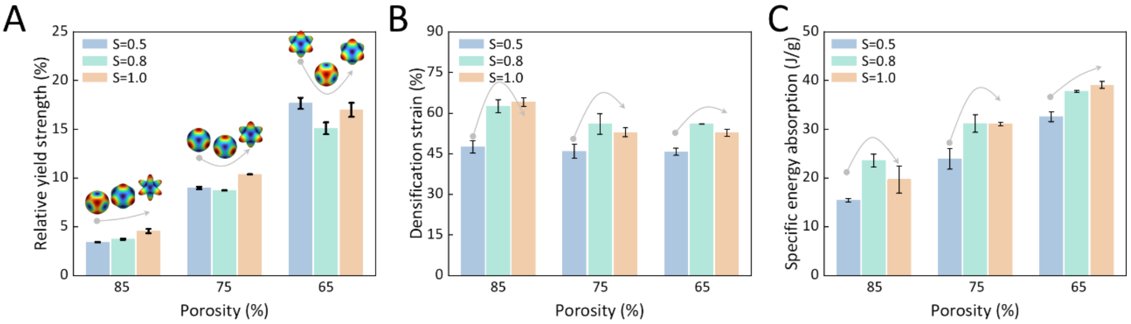

Fig. S17. The mechanical properties of biomimetic metamaterials. Comparison of (**A**) Relative yield strength, (**B**) Densification strain, and (**C**) Specific energy absorption of biomimetic metamaterial with different *S*.

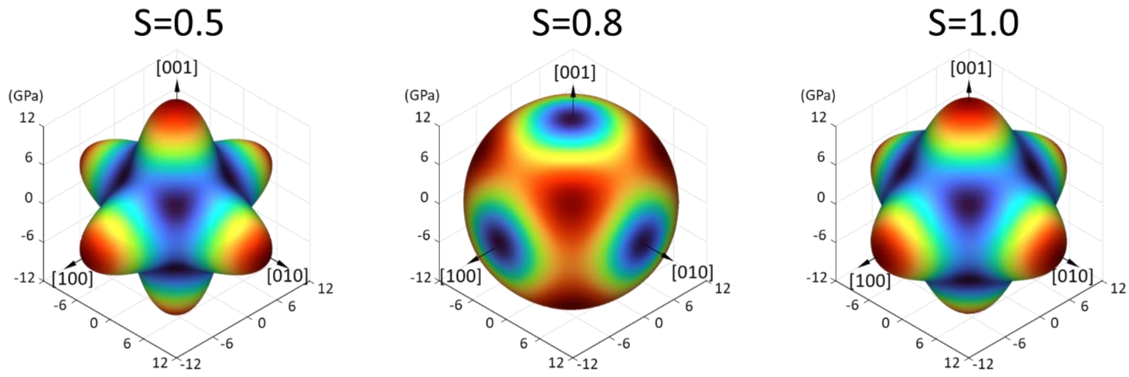

Fig. S18. Unit-cell 3D spatial representations of elastic modulus surfaces of biomimetic metamaterials with 0.5, 0.8, and 1.0 shape factor S.

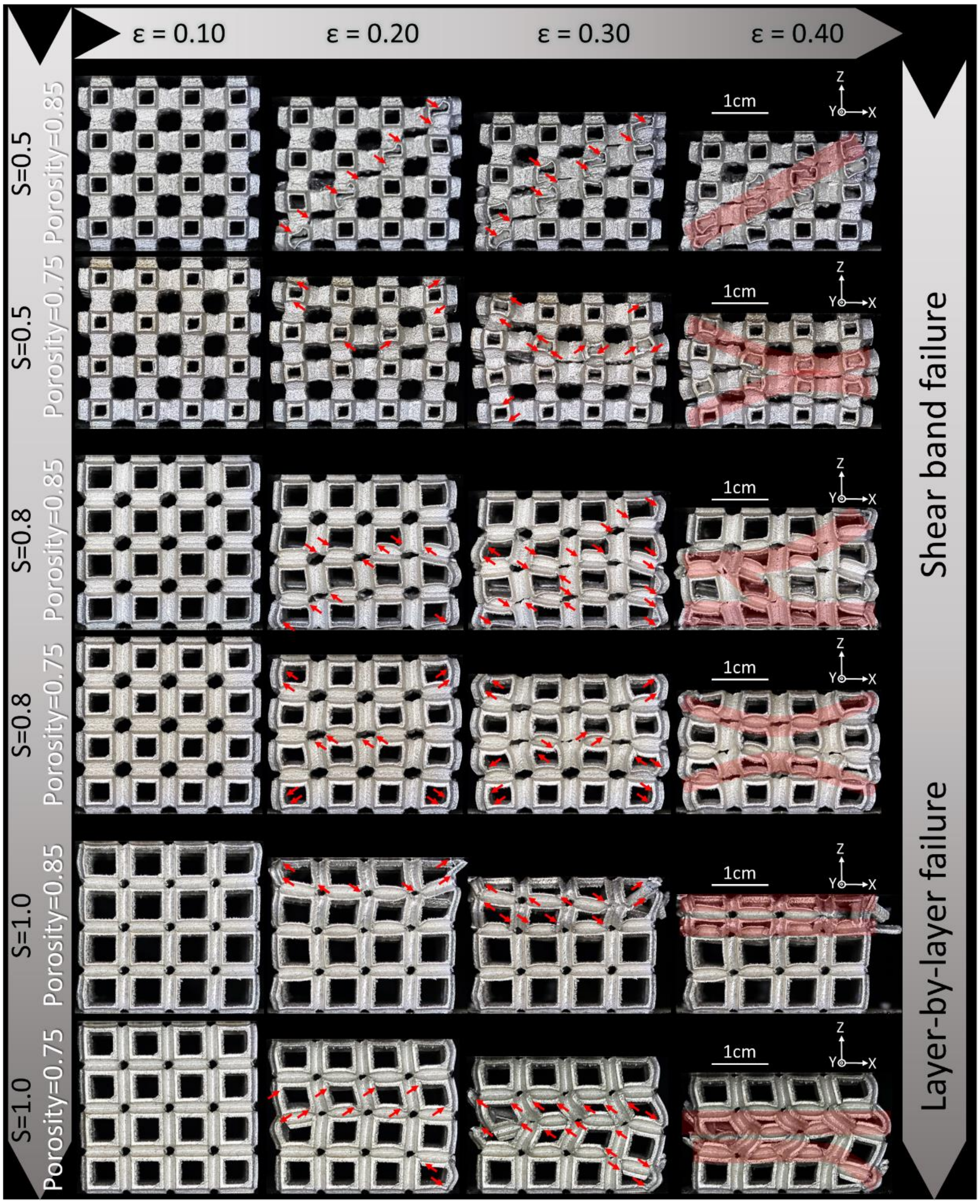

Fig. S19. In-situ compressed images with different porosity and *S*.

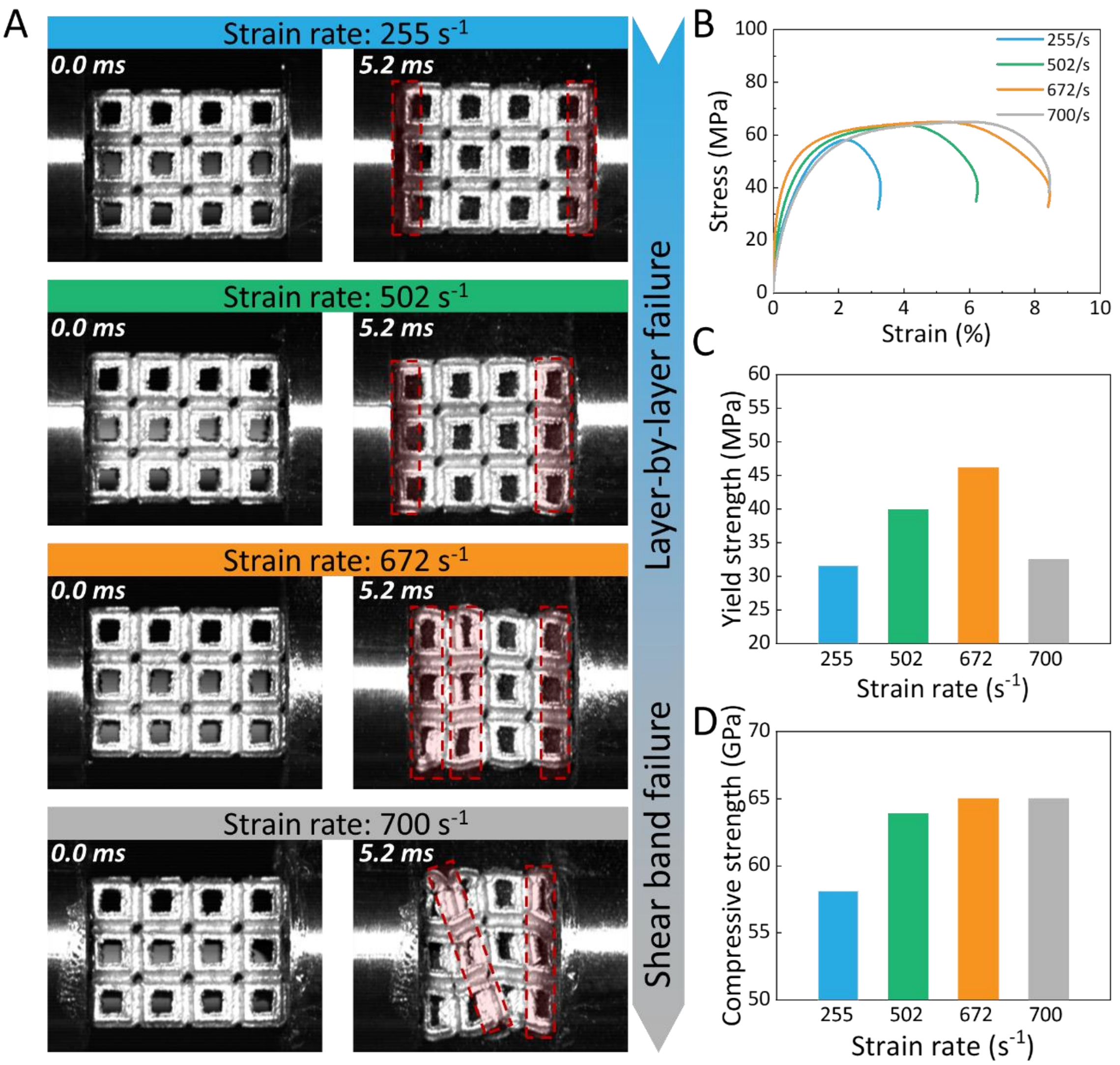

Fig. S20. High-speed photography images and mechanical properties of biomimetic metamaterials under different strain rates. (**A**) High-speed photography images, (**B**) Stress-strain curves, (**C**) Yield strength, and (**D**) Compressive strength of biomimetic metamaterials under four strain rates.

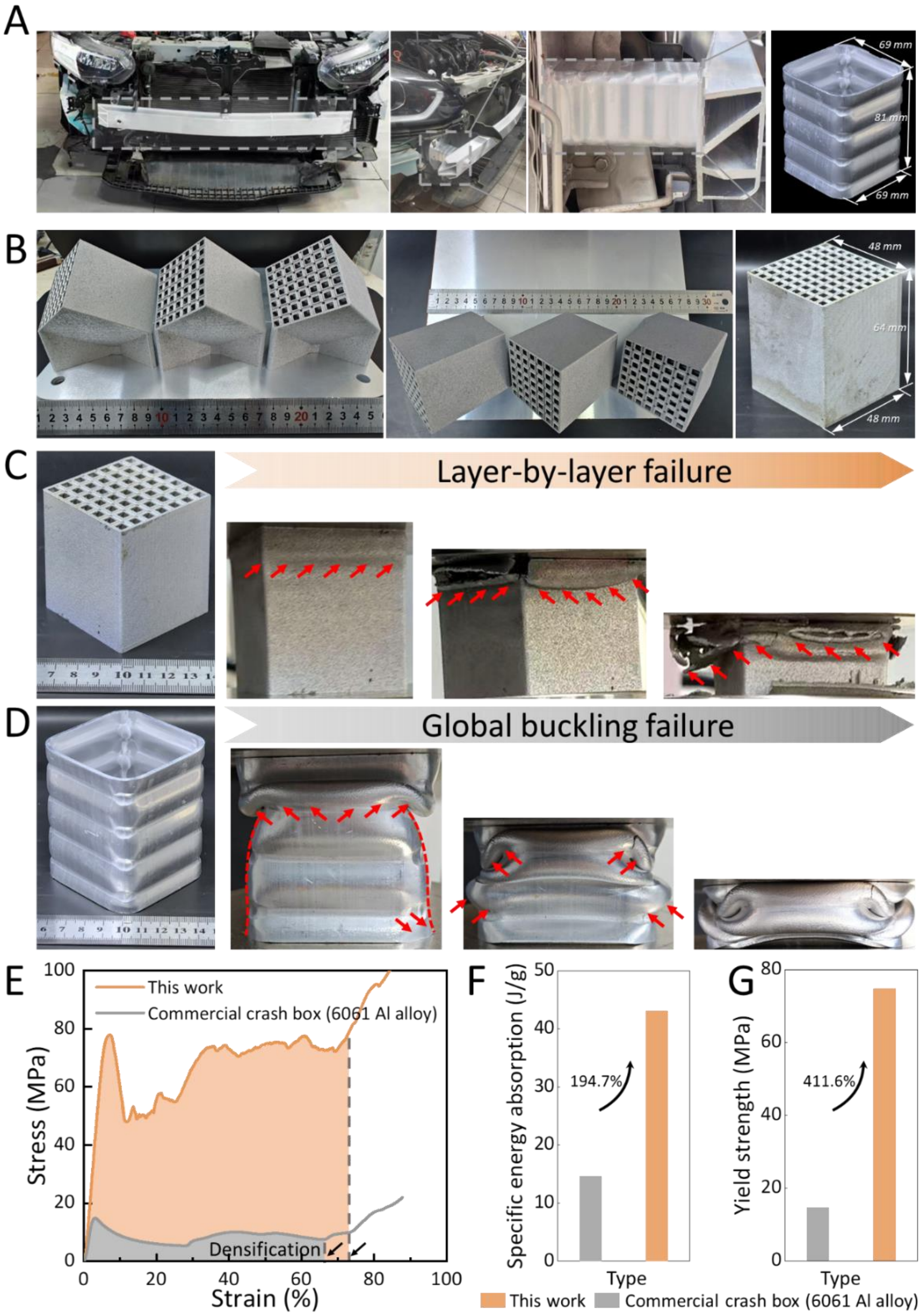

Fig. S21. Quasi-static compression tests of two types of crash boxes. (**A**) A commercial crash box; (**B**) Biomimetic metamaterial-filled crash box (this work); (**C**) In-situ compressed images of biomimetic metamaterial-filled crash box (this work); (**D**) In-situ compressed images of the commercial crash box; (**E**) Stress-strain curves, (**F**) Specific energy absorption, and (**G**) Yield strength of the two crash boxes.

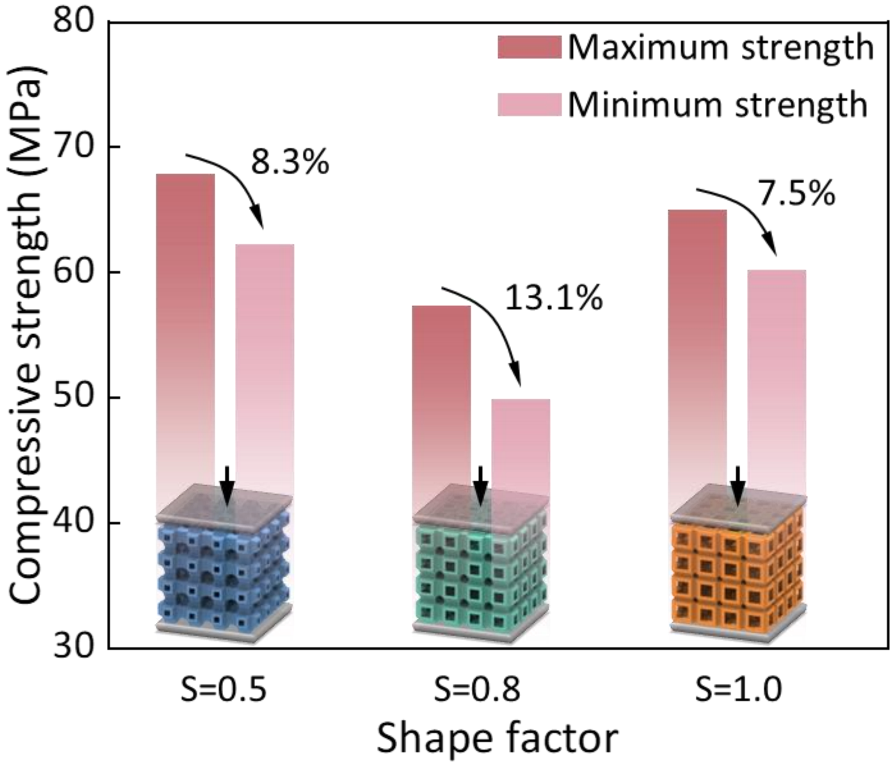

Fig. S22. The maximum/minimum compressive strength of biomimetic metamaterials with 65% porosity of different *S*.

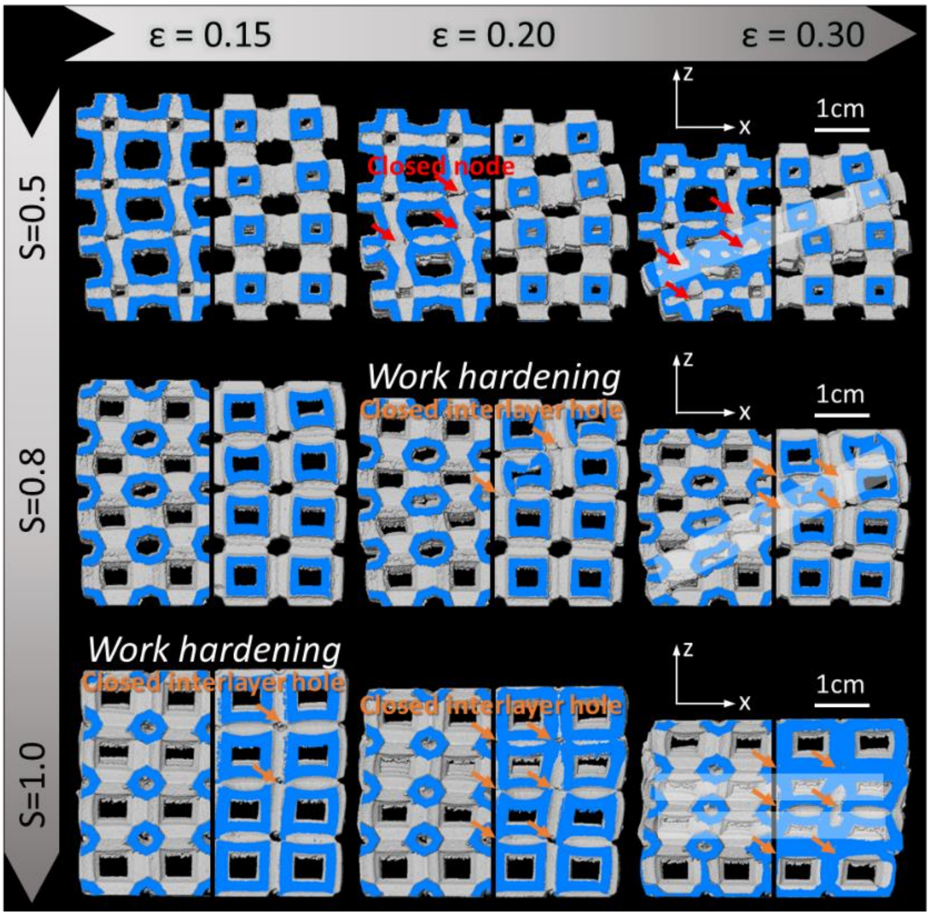

Fig. S23. Micro-CT reconstruction model slices of nodes (left) and struts (right) of biomimetic metamaterials in different strains.

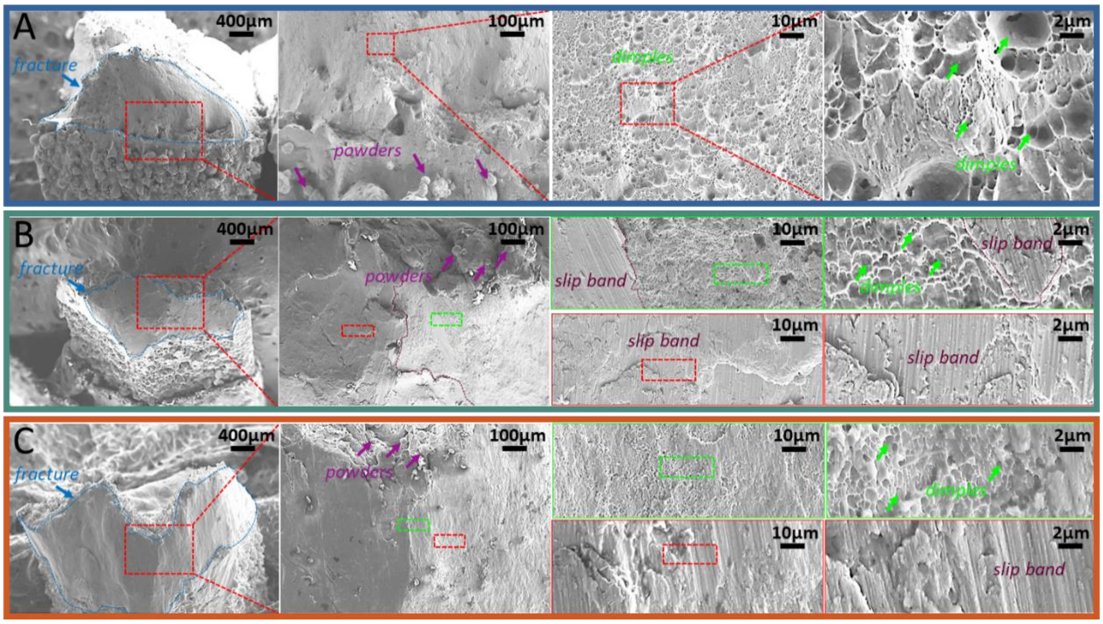

Fig. S24. The fracture morphology of biomimetic metamaterials at different *S* values. (**A**) Fracture morphology at *S*=0.5; (**B**) Fracture morphology at *S*=0.8; (**C**) Fracture morphology at *S*=1.0.

Movie S1. Dynamic impact tests of biomimetic metamaterials under four different strain rates.
